# Habitat Fragmentation in Urbanized Landscapes Favors Bird Species With Darker Plumage

**DOI:** 10.1002/ece3.73417

**Published:** 2026-04-06

**Authors:** Yan Wang, Jie Xie, Yu Liu, Shirui Yu, Shang Sheng, Lingchen Liao, Kai Zhang, Yu Xu

**Affiliations:** ^1^ School of Life Sciences Guizhou Normal University Guiyang China; ^2^ Presbyterian Ladies' College Burwood Australia

**Keywords:** birds, environmental filtering, habitat fragmentation, plumage coloration, species sorting, urbanization

## Abstract

Plumage coloration is an important trait influencing signaling, communication, and ecological adaptation in birds. While some studies have documented that urbanization favors bird species with darker plumage, the specific filtering effects of habitat fragmentation, a common structural consequence of urbanization, remain largely unexplored. We examined how habitat fragmentation favored bird species with particular achromatic plumage color, based on 82 breeding bird species recorded across 30 remnant woodlot patches within a rapidly urbanizing landscape in southwest China between 2017 and 2024. We calculated assemblage‐level achromatic plumage color from species occurrences and species‐level whole‐body mean color values. As hypothesized, assemblages in smaller patches and patches with higher edge proportions had darker plumage when accounting for species richness. This pattern was slightly more pronounced in males than in females. Contrary to our expectations, assemblages in patches with lower cover percentage of woodlands and higher percentage of built‐up areas surrounding patches were not lighter. These results suggest that darker species, especially those with darker males, are more likely to persist in highly fragmented habitats. By interpreting plumage coloration in relation to potential advantages of camouflage, boldness, and pollution resistance, this study provides insights into trait‐based species sorting in urbanized landscapes.

## Graphical abstract

Using long‐term breeding bird survey data from 30 remnant woodlot patches within a rapidly urbanizing landscape in southwest China, we demonstrate that species with darker plumage are more likely to persist in highly fragmented habitats. The pattern is slightly more pronounced in males than in females. These findings provide valuable insights into trait‐based community assembly in urban landscapes.

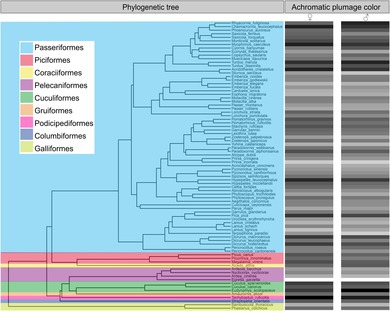

## 1 Introduction

Urban areas are characterized by pervasive human disturbance and extensive impervious surfaces, as well as other environmental stressors such as pollution, artificial light, and increased temperature, which can act as filters to species (Chace and Walsh 2006; Shanahan et al. 2013). This environmental filtering allows the persistence of species with specific life‐history traits, suggesting non‐random community assembly shaped by trait–environment associations in urban environments (Croci et al. 2008; Grimm et al. 2008; Aronson et al. 2016; Salmón et al. 2023).

For birds, plumage coloration is a trait of major importance, shaping signaling, communication, and ecological adaptation (Dale et al. 2015). There are over 11,000 bird species globally, which demonstrate achromatic variation from white to black, through regulated melanin deposition (Delhey 2018). While rising temperatures in urban environments due to urban heat island effects may select species with lighter plumage (Kreling 2023), which reflect more solar radiation and keep body cooler (Roulin 2014; Delhey 2019; Delhey et al. 2023), empirical studies across multiple biogeographic regions have documented that urban environments tend to favor species with darker plumage (Leveau 2019; Leveau 2021). From an ecological perspective, darker plumage in urban environments may provide crypsis against gray impervious substrates (e.g., concrete and asphalt surfaces), potentially reducing detectability by humans and predators (Leveau 2019; Leveau and Ibáñez 2022; Turak et al. 2022), even though predation pressure from natural predators is generally lower in urban areas (Vincze et al. 2017). In terms of behavioral syndromes, darker plumage is likely linked to boldness and exploratory behavior through pleiotropic effects of the melanocortin system (Ducrest et al. 2008; Jiménez et al. 2024). Moreover, urbanization may drive selection for species with darker plumage via physiological mechanisms linked to resource‐, parasite‐, or pollutant‐mediated pressures (Leveau 2019; Turak et al. 2022). Empirical evidence has documented physiological correlates of melanin‐based plumage, including associations with cholesterol metabolism and energy allocation (Arcila et al. 2025), immune function and parasite resistance (Aouissi et al. 2021; Kamiński et al. 2025), and detoxification of heavy metals (e.g., Pb) (Chatelain et al. 2014, 2016).

Habitat fragmentation is a common structural consequence of urbanization, driven by processes such as land conversion, infrastructure expansion, and altered disturbance regimes (Johnson and Munshi‐South 2017). This transformation reshapes natural ecosystems to smaller, discrete habitat patches embedded within inhospitable matrices like impervious surfaces (Johnson and Munshi‐South 2017; Diamond and Martin 2021). Crucially, these fragments are typified by disproportionately high edge‐to‐core ratios (Fahrig 2003), intensifying the ecological stressors associated with edge habitats. Such edge environments, adjacent to gray impervious surfaces, are subjected to stressors such as intensified human disturbance, elevated predation risk, especially from human‐associated predators (e.g., free‐roaming cats and dogs), or potential contamination by toxic metals, which could impose significant constraints on species persistence (Fernández‐Juricic 2002; González‐Oreja et al. 2012; Wang et al. 2013; Tai et al. 2022). However, while existing studies have broadly examined the relationship between urbanization and avian plumage coloration (e.g., Leveau 2019; Leveau and Kopp 2024; Leveau and Ibáñez 2022; Turak et al. 2022; Yu et al. 2024; Ibáñez‐Álamo et al. 2025), few studies have explicitly explored how habitat fragmentation acts as an environmental filter, favoring species with particular plumage color (Leveau and Kopp 2024).

In this study, we investigated breeding birds in 30 remnant woodlot patches within an urban landscape of southwest China, to test the following two hypotheses:

(H1) Bird assemblages in smaller patches, as well as those in patches with a higher proportion of edges, may be dominated by species with darker plumage, potentially reflecting environmental filtering associated with urban stressors, such as intensified human disturbance, elevated predation risk, or increased exposure to chemical pollutants (Leveau 2019; Turak et al. 2022; Kreling 2023; Ibáñez‐Álamo et al. 2025).

In contrast, (H2) bird assemblages in patches surrounded by a lower proportion of woodlands and a higher proportion of built‐up areas, landscape contexts typically associated with higher surface or air temperatures (Arnfield 2003; Roth et al. 2021), may be dominated by species with lighter plumage, potentially reflecting environmental filtering related to thermoregulatory considerations (Kreling 2023; Ibáñez‐Álamo et al. 2025).

## 2 Materials and Methods

### 2.1 Study Area

**FIGURE 1:**
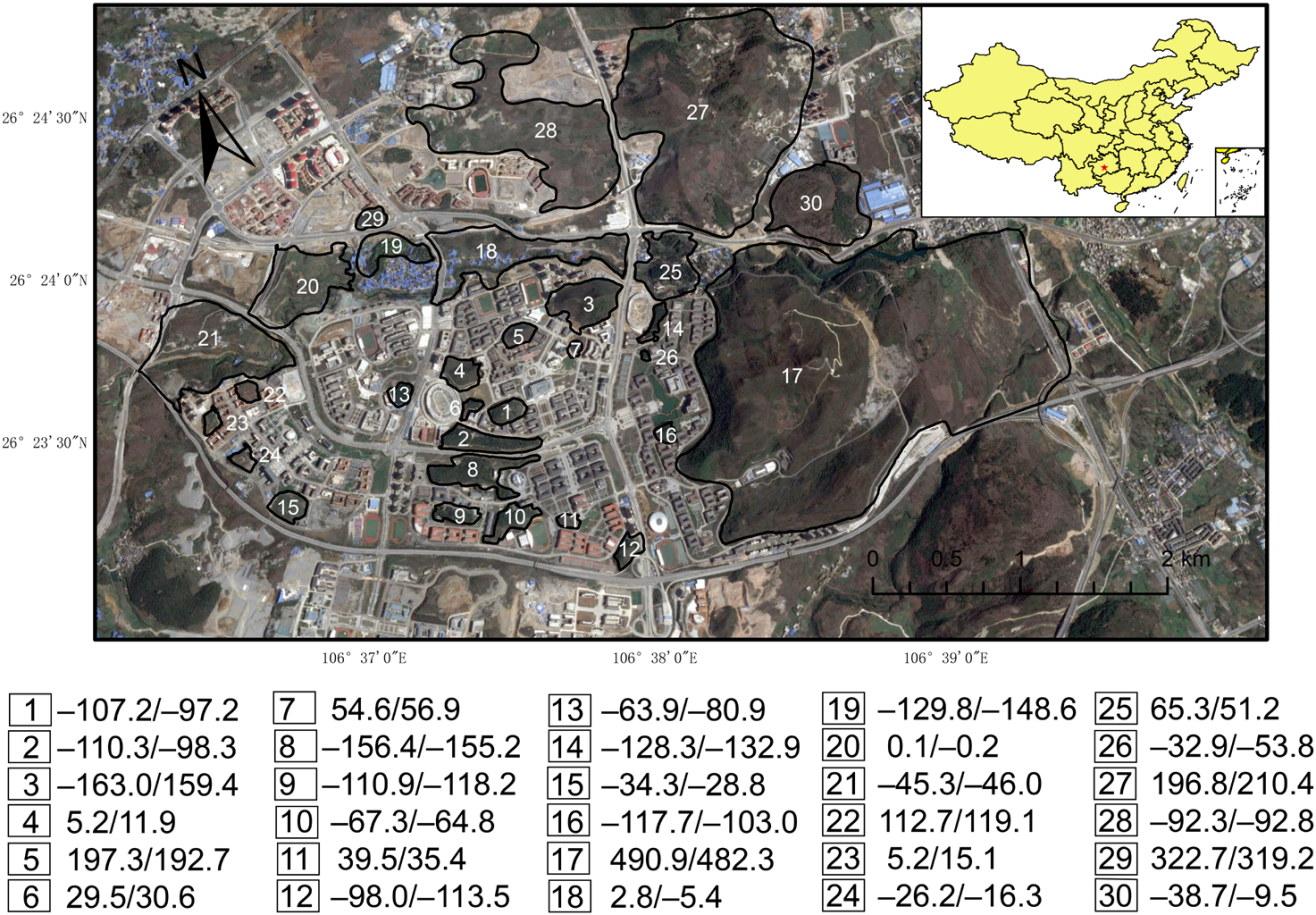
Map illustrating the location of Huaxi University Town, Guizhou Province, southwest China, and the spatial distribution of 30 remnant woodlots (numbered 1–30) overlaid on Google satellite imagery. Below the map, residual values of achromatic plumage color, accounting for species richness, are presented for female and male birds in each patch, with higher values indicating lighter plumage.

This study was conducted in Huaxi University Town (26°23′–26°25′ N, 106°36′–106°40′ E), located in Guizhou Province, southwest China (Figure 1). The region lies at an elevation of 1200 m and is characterized by typical karst landforms, a subtropical humid temperate climate, an average annual temperature of approximately 15°C, and an annual rainfall of around 1100 mm (Zheng et al. 2021; Liu et al. 2024; Zhu et al. 2024). Prior to the town's construction in 2009, the area consisted of natural woodlots interspersed with croplands. Subsequent urban development, including the construction of roads and buildings, has fragmented these habitats. For this survey, we selected 30 remaining woodlot patches (Figure 1). The landscapes of these patches remained largely unchanged during the survey period, with only minor changes from the addition of a few buildings at the edges of three larger patches in recent years. These patches were primarily composed of woodlands, with additional habitats including scrublands, grasslands, wetlands, and croplands. The dominant tree species included Masson's Pine *Pinus massoniana* , Sharptooth Oriental White Oak *Quercus aliena* var. *acutiserrata*, and Platycarya *Platycarya strobilacea* , while shrubs were represented by Chinese Firethorn *Pyracantha fortuneana* , Masuri Berry *Coriaria nepalensis*, and Elderflower Rose *Rosa cymosa* (Cao 2021).

We obtained high‐resolution satellite imagery of the study area from Google Earth and delineated patch boundaries in ArcGIS 10.7. For each patch, we measured patch area (ranging from 0.3 to 290.4 ha) and shape index (SI, ranging from 109.5 to 201.1; Table S1). SI reflects the complexity of patch shape and serves as a proxy for edge effects. It is calculated as:

$$\mathrm{SI}=\mathrm{Pe}/\left[2\times {\left(\mathrm{\pi A}\right)}^{0.5}\right]$$

where *Pe* is patch perimeter, and *A* is patch area. Higher SI values indicate more irregular patch shapes and stronger potential edge effects.

We downloaded 10‐m resolution land cover data from the WorldCover 2021 v.200 product (https://esa‐worldcover.org/en), which provides 11 primary land use categories with an overall accuracy of 76.7% (Zanaga et al. 2022). To minimize potential classification errors, we manually verified and corrected the data using Google Earth and our field observations. Then, we measured the cover percentage of woodlands within patches (ranging from 3.9% to 96.6%), and the percentage of built‐up areas within a 500 m buffer surrounding patches (ranging from 14.2% to 58.5%; Table S1). Pearson correlation analyses showed that the percentage of built‐up areas surrounding patches was negatively correlated with patch area (*r* = −0.51, *p* = 0.004), indicating that increasing urbanization intensity is primarily associated with reductions in patch area, a key component of habitat fragmentation, in the study landscape. However, the percentage of built‐up areas showed no significant relationship with SI (*r* = 0.03, *p* = 0.880), suggesting that patch shape complexity may be influenced by other factors, such as land division patterns (e.g., road networks and infrastructure layout), rather than urbanization intensity per se.

### 2.2 Field Survey and Data Collection

Bird surveys were conducted using line‐transect methods (Zheng et al. 2021; Liu et al. 2024; Zhu et al. 2024; Xie et al. 2025). In the largest patch (290.4 ha), four transects were laid out, while each of the remaining patches had one transect. Transect lengths ranged from 82.5 to 7073.7 m, approximately proportional to patch area, to reduce sampling bias (Schoereder et al. 2004). Surveys were carried out annually between 2017 and 2024 during the bird breeding seasons (April to August). Observations took place in favorable weather conditions, excluding rain, wind, and fog. Each day, surveys were conducted from 07:00 to 10:00 and 16:00 to 18:00, coinciding with peak activity periods for most bird species. Two observers walked transects at a constant pace (approximately 1.5 km/h), recording all birds detected visually or acoustically within 50 m on either side. To minimize interference from adjacent patches, only calls and songs clearly attributable to the surveyed patch were included. Species identification followed MacKinnon and Phillipps (2000) and Viney et al. (2017). Across the study, 18 survey rounds were completed: three each in 2017–2018, four in 2019, one in 2020, two in 2021, one in 2022, three in 2023, and one in 2024.

### 2.3 Achromatic Plumage Color Data

We obtained data on species‐level achromatic plumage color from Delhey et al. (2021), measured on a scale from 0 (black) to 100 (white). These values were quantified through the Handbook of the Birds of the World plates, which includes approximately 20,000 digitized bird images representing over 10,000 species (Images depicting incomplete plumage, juveniles, or non‐breeding plumages were excluded from the analysis). For each image, RGB values for each pixel across the entire visible plumage (including both dorsal and ventral surfaces as depicted in the illustration) were extracted and converted into the CIELAB color space, which allows for achromatic dimensions, primarily influenced by melanin deposition, to be analyzed independently from chromatic dimensions (i.e., hue and saturation). These values were then averaged across all pixels to obtain a single value for each species and sex. Although the colors in the plates are based on human perception rather than avian vision, previous studies have demonstrated robust correlations between these color data and reflectance spectrometry results modeled for avian vision (Bergeron and Fuller 2018; Delhey et al. 2023), validating their use in similar work (Delhey et al. 2023; Wu et al. 2024; Yu et al. 2024; Ibáñez‐Álamo et al. 2025).

### 2.4 Statistical Analyses

We aggregated 18‐round surveys to assess bird assemblages based on species presence–absence across patches, including only species that breed in the study area (i.e., resident and summer‐visiting breeding species). Birds recorded only once and high‐flying species that are less likely to utilize woodlot patches (e.g., raptors, swallows, and swifts) were excluded to avoid introducing noise that could compromise assemblage inference. Using species occurrences and species‐level mean plumage color values, we calculated assemblage‐level achromatic color for each patch and used linear regression models to examine its relationship with landscape and habitat metrics, including patch area, SI, the cover percentage of woodlands within patches, and the percentage of built‐up areas within a 500 m buffer surrounding patches. Since assemblage‐level achromatic color increased with species richness, we first regressed achromatic color against species richness and extracted the resulting residuals as the dependent variable in subsequent analyses. We constructed models incorporating all combinations of explanatory variables and evaluated their performance using the small‐sample corrected Akaike Information Criterion (*AICc*). The model with the lowest *AICc* was considered the best‐supported when the difference in *AICc* values (*ΔAIC*_*c*_) between it and second‐ranked model exceeded 2 (Burnham and Anderson 2002). Otherwise, we performed model averaging based on conditional averaging on the model set with cumulative Akaike weights ≤ 0.95 (Grueber et al. 2011; Symonds and Moussalli 2011), and calculated standardized regression coefficients using partial standard deviation scaling (Cade 2015).

**FIGURE 2:**
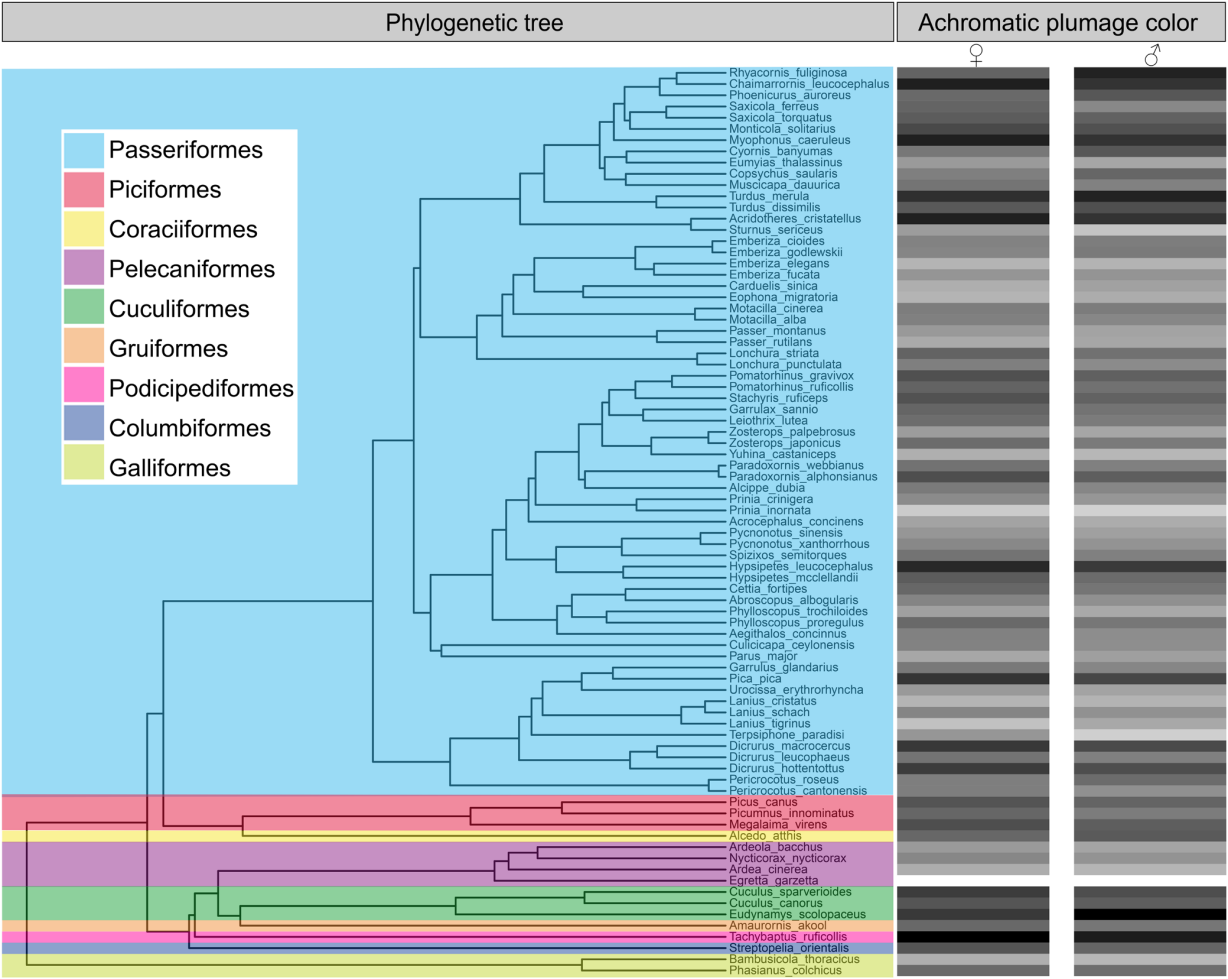
Achromatic plumage color of bird species recorded, showed as heatmaps aligned to the tips of the phylogenetic tree. The grayscale tone represents the whole‐body mean color value (scale: 0 [black] to 100 [white]; Table S3) for each species and sex (female, ♀; male, ♂), compiled from Delhey et al. (2021). The Spotted Dove *Stigmatopelia chinensis* was excluded due to the lack of data on achromatic plumage color. The phylogenetic tree was obtained by randomly sampling 5000 trees from the global avian phylogenetic dataset from BirdTree (http://birdtree.org) under the option of ‘Hackett All Species: A set of 10 000 trees with 9993 OTUs each’ (Jetz et al. 2012) and summarizing them into a Maximum Clade Credibility (MCC) tree with mean node heights using TreeAnnotator v1.10.5 from the BEAST software.

To account for potential sexual dimorphism in plumage color (Figure 2), we analyzed the data separately for females and males (Ibáñez‐Álamo et al. 2025). Furthermore, to examine whether the observed patterns were influenced by non‐passerine species, which may require larger territories, or by migratory species, which can present different plumage coloration (Delhey et al. 2021), we further restricted the analysis to passerine and sedentary species (i.e., excluding summer‐visiting breeding species), respectively. Given that the short distances between some patches may allow inter‐patch movement of some species, we also conducted spatial autoregressive models to assess the robustness of our results. Only the significant predictors from the prior best‐fitting or averaged models included, and a spatial weight matrix was constructed using a threshold of 50 m inter‐patch distance to account for potential spatial autocorrelation (Xie et al. 2025; Liu et al. 2026).

We performed the analyses and visualized data using R version 4.3.2 with packages ‘MuMIn’ (Bartoń 2024), ‘spatialreg’ (Bivand et al. 2021), and ‘visreg’ (Breheny and Burchett 2017).

## 3 Results

We recorded 82 bird species, with the number of species per patch ranging from 14 to 62 (Table S2). Species rarefaction curves for each patch indicated that the sampling efforts were generally sufficient to capture the majority of species present (Figure S1).

**FIGURE 3:**
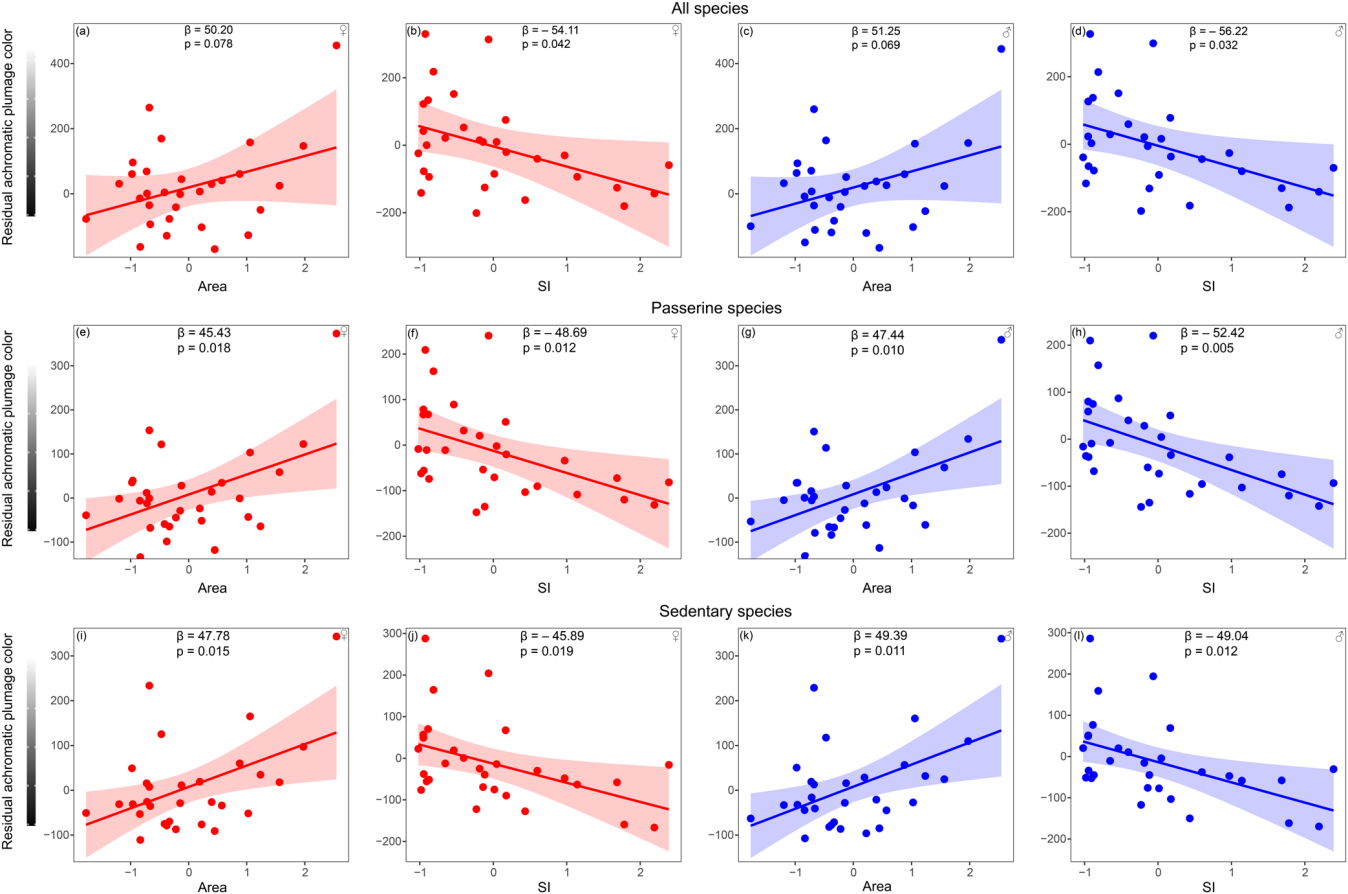
Partial residual plots showing the relationships between residual achromatic plumage color (accounting for species richness) and (a, c, e, g, i, k) patch area (log‐transformed and standardized) and (b, d, f, h, j, l) shape index (SI, log‐transformed and standardized) for females (♀) and males (♂). Panels (a–d) show results for all species, (e–h) for passerine species only, and (i–l) for sedentary species only. Plots for all species are based on a linear model including the same variables as those in the final averaged model subset, while plots for passerine and sedentary species are based on their respective single best‐supported models. *β* represents the regression coefficient estimate, and shaded areas represent 95% confidence intervals. For more statistical details, see Table S5.

Achromatic plumage color showed a significant interspecific variation with a spectrum ranging from 34.6 to 82.6 (Figure 2). Residual achromatic plumage color of bird assemblages (accounting for species richness) varied significantly among patches, ranging from −163.0 to 490.9 (Figure 1). No single model with Δ*AICc* greater than 2 explained the variation in residual achromatic plumage color (Table S4); model‐averaging results showed a positive relationship between achromatic color and patch area (Figure 3a,c), indicating that assemblages in larger patches had lighter plumage while those in smaller patches had darker plumage. In addition, there was a negative relationship between achromatic color and SI (Figure 3b,d), suggesting that assemblages in patches with higher SI had darker plumage while those with lower SI had lighter plumage. The effects were similar in both females (Figure 3a,b) and males (Figure 3c,d), but they were slightly more pronounced in males, as indicated by the regression coefficient estimates. Neither the cover percentage of woodlands within patches nor the percentage of built‐up areas within a 500 m buffer surrounding patches had significant effects on residual achromatic color for either sex (see Table S5).

For analyses restricted to passerine species and sedentary species, the best‐supported models (with *ΔAICc* lower than 2; Table S4) revealed patterns consistent with those obtained using all species (Figure 3e–l). The effects of these explanatory variables also remained similar in the spatial autoregressive models (Table S6), indicating that our results are robust.

## 4 Discussion

As expected, we found that bird assemblages in smaller patches and patches with higher edge proportions had darker plumage. The results suggest that habitat fragmentation in the urbanized landscape, including both habitat loss and edge effects, may favor bird species with darker plumage. In contrast to other traits, such as body size, habitat specificity, clutch size, trophic level, territoriality, or flocking tendency, which show little evidence of filtering species in our study system (Zhu et al. 2024). Our findings indicate that plumage coloration may serve as a potentially more sensitive indicator of species sorting.

Urbanization transforms natural ecosystems into discrete habitat patches embedded within inhospitable matrices, such as impervious surfaces (e.g., concrete, asphalt). Although predation rates due to natural predators tend to be lower in urban environments (Vincze et al. 2017), smaller habitat patches with higher edge proportions are more susceptible to human disturbance and predation from human‐induced predators such as free‐roaming cats and dogs (Fernández‐Juricic 2002; González‐Oreja et al. 2012; Wang et al. 2013; Tai et al. 2022). During our fieldwork, we found instances where birds such as the Oriental Turtle‐Dove *Streptopelia orientalis* and White‐browed Laughingthrush *Garrulax sannio* , especially those at patch edges or in croplands, were harassed or injured by humans or free‐roaming cats or dogs. Light‐plumaged species may be more visible against impervious surfaces at patch edges, potentially increasing their detectability and vulnerability to human and predator interference (Leveau 2019; Turak et al. 2022; Kreling 2023; Ibáñez‐Álamo et al. 2025). Conversely, species with darker plumage may improve camouflage against gray impervious substrates and experience improved survival.

In addition, species with darker plumage occupying smaller habitat patches and particularly those with higher edge proportions may involve the pleiotropic effects of the melanocortin system. Melanin‐based coloration is functionally linked to a suite of behavioral and physiological traits, including stress tolerance and boldness (Ducrest et al. 2008). More melanic individuals often show increased exploratory behavior and reduced stress responsiveness, which may enhance their ability to cope with the strong edge effects of small and highly fragmented patches, therefore facilitating the persistence of darker species in these patches. Moreover, the observed patterns may confer potential advantages under localized pollution exposure. In our study area, although industrial activity is limited, patch edges, particularly those adjacent to major traffic corridors, are more likely to experience localized deposition of traffic‐derived metals (e.g., Pb and Cd). Species with melanin‐based plumage may partially sequester these metals in their feathers, potentially reducing systemic exposure (Chatelain et al. 2014, 2016). However, the magnitude of this effect likely varies spatially across urban landscapes depending on local traffic density, human activity, and patch configuration.

The observed effect was slightly more pronounced in male birds compared to their female counterparts. Typically, sexual selection drives males to display more conspicuous ornamentation, while females maintain cryptic coloration (Dale et al. 2015; Delhey et al. 2023). However, urbanization may weaken the strength of sexual selection for coloration signals, especially due to the influence of disturbance–predation pressure on plumage coloration (Croci et al. 2008; Iglesias‐Carrasco et al. 2019; Kreling 2023). Given that male birds may face greater human disturbance and higher predation risk due to engaging in frequent foraging excursions into open spaces or edge habitats, where they may receive supplemental food from human activities (Zhu et al. 2022, 2024), in contrast to nesting females, this shift in survival pressure is more likely to select for species with darker males, enhancing crypsis during foraging.

We do not interpret our findings in terms of physiological mechanisms linked to resource‐ or parasite‐mediated pressures (Aouissi et al. 2021; Arcila et al. 2025; Kamiński et al. 2025) because human food availability and parasite pressure are not necessarily higher in smaller patches with higher edge proportions and may instead be strongly influenced by local context, such as patch‐specific management, surrounding land use, or microhabitat conditions. Contrary to our hypothesis, assemblages in patches with a lower percentage of woodlands or a higher percentage of built‐up areas surrounding patches were not lighter. This suggests environmental filtering related to thermoregulatory considerations is weak or absent in our study system. Although patches with lower woodland cover or higher surrounding built‐up areas may experience elevated surface or air temperatures (Arnfield 2003; Roth et al. 2021), the thermoregulatory benefits of lighter plumage probably do not outweigh the potential advantages of darker plumage (Kreling 2023). It is also possible that widespread, habitat‐tolerant passerines could potentially obscure subtle filtering effects. However, we were unable to evaluate this possibility because non‐passerine species were much less numerous (17 species), with only one or two species present in many patches, which could limit statistical power.

We acknowledge that short distances between some patches may allow movement for some species and could potentially violate assumptions of data independence, but spatial autoregressive models yielded results consistent with our main analyses, supporting the robustness of our findings. We thus argue that any potential non‐independence is likely limited to a small number of widespread or highly mobile species that occupy larger spatial extents. In contrast, many behaviorally sensitive species, particularly those with strong site fidelity or territoriality, are likely to remain patch‐bound during the breeding season. This interpretation is supported by our previous work demonstrating high species compositional dissimilarity among patches (Liu et al. 2024), indicating that species sorting operates strongly across patches. Notably, plumage coloration rather than other traits such as body size, habitat specificity, clutch size, trophic level, territoriality, or flocking tendency (Zhu et al. 2024) may represent a more sensitive indicator of species sorting in our study system. One possible explanation is that these traits, which are primarily linked to resource use, reproduction, and competition, can show delayed responses to habitat fragmentation due to extinction debt (Hylander and Ehrlen 2013; Zhu et al. 2024), particularly given the relatively recent establishment of the urban landscape and the availability of supplemental food from nearby human activities (Zhu et al. 2022, 2024).

To conclude, our findings suggest that plumage coloration may serve as a useful indicator of trait‐based bird species sorting under habitat fragmentation in urbanized landscapes. Darker species, especially those with darker males, are more likely to persist in highly fragmented habitats, potentially due to non‐exclusive advantages such as enhanced camouflage, increased boldness, or increased pollution resistance, which could hypothetically influence their performance in such environments. It should be noted, however, that camouflage or visibility effects likely depend on both background luminance and body regions of plumage coloration (e.g., dorsal vs. ventral surfaces) (Hill and McGraw 2006; Gomez and Théry 2007; Delhey 2019; de la Torre et al. 2024). In addition, both predation pressure and pollution exposure are likely to vary spatially and context‐dependently across urban landscapes (Novoa et al. 2025). Further studies that explicitly incorporate these factors, as well as chromatic plumage coloration, which may provide complementary insights (e.g., Delhey et al. 2023; Ibáñez‐Álamo et al. 2025), are needed to further refine these inferences. We cannot determine whether intraspecific variation in plumage coloration occurs in the study system because individual‐level plumage data were not collected. Plastic or evolutionary responses within species may nevertheless occur, particularly over longer temporal scales (Kreling 2023), warranting future studies based on individual‐level plumage measurements.

## Author Contributions

**Yan Wang:** data curation (lead), formal analysis (lead), investigation (equal), methodology (supporting), software (lead), validation (lead), visualization (lead), writing – original draft (lead). **Jie Xie:** data curation (supporting), investigation (equal). **Yu Liu:** investigation (equal). **Shirui Yu:** investigation (equal). **Shang Sheng:** investigation (equal). **Lingchen Liao:** investigation (equal). **Kai Zhang:** investigation (equal), methodology (supporting). **Yu Xu:** conceptualization (lead), data curation (supporting), funding acquisition (lead), investigation (equal), methodology (lead), project administration (lead), supervision (lead), validation (supporting), writing – review and editing (lead).

## Funding

This work was supported by the National Natural Science Foundation of China (32270540), the Joint Fund of the National Natural Science Foundation of China and the Karst Science Research Center of Guizhou Province (U1812401), and the Science and Technology Program of Guizhou Province (ZK[2021]098).

## Conflicts of Interest

The authors declare no conflicts of interest.

## Supporting information


**Table S1:** Landscape and habitat characteristics of 30 patches.
**Table S2:** Bird species‐by‐patch presence (1)/absence (0) matrix across 30 woodlot patches. Species taxonomy and nomenclature are based on BirdTree. The Spotted Dove *Stigmatopelia chinensis* was excluded from the analysis due to the lack of data on achromatic plumage color.
**Table S3:** Achromatic plumage color values of bird species recorded, shown separately for both sexes. The values represent the whole‐body mean color of each species, compiled from Delhey et al. (2021). Species taxonomy and nomenclature are based on BirdTree. The Spotted Dove *Streptopelia chinensis* was excluded due to the lack of data on achromatic plumage color.
**Table S4:** Model set with cumulative Akaike weights ≤ 0.95 examining the relationships between residual achromatic plumage color (accounting for species richness) and landscape and habitat metrics for female and male birds across all, passerine, and sedentary species. Metrics include patch area, shape index (SI), the cover percentage of woodlands within patches (Woodlands), and the percentage of built‐up areas within a 500 m buffer surrounding patches (Built_up). Patch area and SI were log‐transformed, and all variables were centered and standardized. The statistics include the number of parameters (*K*), log‐likelihood value (*logLik*), Akaike's information criterion corrected for small sample size (*AICc*), difference in *AICc* relative to the minimum *AICc* (Δ*AICc*), and Akaike weights (*w*_*i*_).
**Table S5:** Model‐averaging results (from the model set with cumulative Akaike weights ≤ 0.95) for all species, and best‐supported model results for passerine and sedentary species, examining the variation in residual achromatic plumage color (accounting for species richness) in relation to landscape and habitat metrics for female and male birds. Metrics included patch area, shape index (SI), the cover percentage of woodlands within patches (Woodlands), and the percentage of built‐up areas within a 500 m buffer surrounding habitat patches (Built_up). Patch area and SI were log‐transformed, and all variables were centered and standardized. Statistics include regression coefficient estimates, standard errors (SE), *t* values, and *p* values.
**Table S6:** Results of spatial autocorrelation models examining the variation in residual achromatic plumage color (accounting for species richness) in relation to landscape and habitat metrics for female and male birds across all, passerine and sedentary species. Only the significant predictors from the prior best‐fitting or averaged models were included. Statistics include coefficient estimates, standard errors (SE), *v* values, and *p* values.
**Figure S1:** Sample‐based curves of bird species in 30 patches.

## Data Availability

The data files and code to reproduce the results of this study are available at https://figshare.com/s/9a7728da710b3d834ee4.

## References

[ece373417-bib-0001] Aouissi, H. A. , M. Ababsa , A. Gaagai , Z. Bouslama , Y. Farhi , and H. Chenchouni . 2021. “Does Melanin‐Based Plumage Coloration Reflect Health Status of Free‐Living Birds in Urban Environments?” Avian Research 12: 45. 10.1186/s40657-021-00280-7.

[ece373417-bib-0002] Arcila, J. , I. Peña‐Villalobos , C. B. Muñoz‐Pacheco , et al. 2025. “Urbanization's Hidden Influence: Linking Landscape Alterations and Feather Coloration With Pigeon's Cholesterol Levels.” Environmental Research 271: 121115. 10.1016/j.envres.2025.121115. 39952457

[ece373417-bib-0003] Arnfield, A. J. 2003. “Two Decades of Urban Climate Research: A Review of Turbulence, Exchanges of Energy and Water, and the Urban Heat Island.” International Journal of Climatology 23: 1–26. 10.1002/joc.859.

[ece373417-bib-0004] Aronson, M. F. , C. H. Nilon , C. A. Lepczyk , et al. 2016. “Hierarchical Filters Determine Community Assembly of Urban Species Pools.” Ecology 97: 2952–2963. 10.1002/ecy.1535. 27870023

[ece373417-bib-0005] Bartoń, K. 2024. MuMIn: Multi‐Model Inference (Version 1.48.4). https://cran.r‐project.org/web/packages/MuMIn/.

[ece373417-bib-0006] Bergeron, Z. T. , and R. C. Fuller . 2018. “Using Human Vision to Detect Variation in Avian Coloration: How Bad Is It?” American Naturalist 191: 269–276. 10.1086/695282. 29351010

[ece373417-bib-0007] Bivand, R. , G. Millo , and G. Piras . 2021. “A Review of Software for Spatial Econometrics in R.” Mathematics 9: 1276. 10.3390/math9111276.

[ece373417-bib-0008] Breheny, P. , and W. Burchett . 2017. “Visualization of Regression Models Using Visreg.” R Journal 9: 56–71. 10.32614/RJ-2017-046.

[ece373417-bib-0009] Burnham, K. P. , and D. R. Anderson . 2002. Model Selection and Multimodel Inference: A Practical Information‐Theoretic Approach. Springer.

[ece373417-bib-0010] Cade, B. S. 2015. “Model Averaging and Muddled Multimodel Inferences.” Ecology 96: 2370–2382. 10.1890/14-1639.1. 26594695

[ece373417-bib-0011] Cao, Z. 2021. Study on Diversity and Spatial Distribution Pattern of Woody Plants in Fragmented Woodlots in Huaxi University Town, Guizhou. M.S. Thesis. Guizhou Normal University.

[ece373417-bib-0012] Chace, J. , and J. Walsh . 2006. “Urban Effects on Native Avifauna: A Review.” Landscape and Urban Planning 74: 46–69. 10.1016/j.landurbplan.2004.08.007.

[ece373417-bib-0013] Chatelain, M. , J. Gasparini , and A. Frantz . 2016. “Do Trace Metals Select for Darker Birds in Urban Areas? An Experimental Exposure to Lead and Zinc.” Global Change Biology 22: 2380–2391. 10.1111/gcb.13170. 27282322

[ece373417-bib-0014] Chatelain, M. , J. Gasparini , L. Jacquin , and A. Frantz . 2014. “The Adaptive Function of Melanin‐Based Plumage Coloration to Trace Metals.” Biology Letters 10: 20140164. 10.1098/rsbl.2014.0164. 24671830 PMC3982444

[ece373417-bib-0015] Croci, S. , A. Butet , and P. Clergeau . 2008. “Does Urbanization Filter Birds on the Basis of Their Biological Traits.” Condor 110: 223–240. 10.1525/cond.2008.8409.

[ece373417-bib-0016] Dale, J. , C. J. Dey , K. Delhey , B. Kempenaers , and M. Valcu . 2015. “The Effects of Life History and Sexual Selection on Male and Female Plumage Colouration.” Nature 527: 367–370. 10.1038/nature15509. 26536112

[ece373417-bib-0017] de la Torre, G. M. , V. Aguiar de Souza Penha , and L. T. Manica . 2024. “Differences in Plumage Coloration Between Ventral and Dorsal Regions on Atlantic Forest Birds.” Ibis 167: 765–775. 10.1111/ibi.13383.

[ece373417-bib-0018] Delhey, K. 2018. “Darker Where Cold and Wet: Australian Birds Follow Their Own Version of Gloger's Rule.” Ecography 41: 673–683. 10.1111/ecog.03040.

[ece373417-bib-0019] Delhey, K. 2019. “A Review of Gloger's Rule, an Ecogeographical Rule of Colour: Definitions, Interpretations and Evidence.” Biological Reviews 94: 1294–1316. 10.1111/brv.12503. 30892802

[ece373417-bib-0020] Delhey, K. , J. Dale , M. Valcu , and B. Kempenaers . 2021. “Migratory Birds Are Lighter Coloured.” Current Biology 31: R1511–R1512. 10.1016/j.cub.2021.10.048. 34875236

[ece373417-bib-0022] Delhey, K. , M. Valcu , C. Muck , J. Dale , and B. Kempenaers . 2023. “Evolutionary Predictors of the Specific Colors of Birds.” Proceedings of the National Academy of Sciences 120: e2217692120. 10.1073/pnas.2217692120. PMC10450850 37579151

[ece373417-bib-0023] Diamond, S. E. , and R. A. Martin . 2021. “Evolution in Cities.” Annual Review of Ecology, Evolution, and Systematics 52: 519–540. 10.1146/annurev-ecolsys-012021021402.

[ece373417-bib-0024] Ducrest, A.‐L. , L. Keller , and A. Roulin . 2008. “Pleiotropy in the Melanocortin System, Coloration and Behavioural Syndromes.” Trends in Ecology & Evolution 23: 502–510. 10.1016/j.tree.2008.06.001. 18644658

[ece373417-bib-0025] Fahrig, L. 2003. “Effects of Habitat Fragmentation on Biodiversity.” Annual Review of Ecology, Evolution, and Systematics 34: 487–515. 10.1146/annurev.ecolsys.34.011802.132419.

[ece373417-bib-0026] Fernández‐Juricic, E. 2002. “Can Human Disturbance Promote Nestedness? A Case Study With Breeding Birds in Urban Habitat Fragments.” Oecologia 131: 269–278. 10.1007/s00442-002-0883-y. 28547695

[ece373417-bib-0027] Gomez, D. , and M. Théry . 2007. “Simultaneous Crypsis and Conspicuousness in Color Patterns: Comparative Analysis of a Neotropical Rainforest Bird Community.” American Naturalist 169: S42–S61. 10.1086/510138. 29517929

[ece373417-bib-0028] González‐Oreja, J. A. , A. A. de la Fuente‐Díaz , L. Hernández‐Santín , C. Bonache‐Regidor , and D. Buzo‐Franco . 2012. “Can Human Disturbance Promote Nestedness? Songbirds and Noise in Urban Parks as a Case Study.” Landscape and Urban Planning 104: 9–18. 10.1016/j.landurbplan.2011.09.001.

[ece373417-bib-0029] Grimm, N. B. , S. H. Faeth , N. E. Golubiewski , et al. 2008. “Global Change and the Ecology of Cities.” Science 319: 756–760. 10.1126/science.1150195. 18258902

[ece373417-bib-0030] Grueber, C. E. , S. Nakagawa , R. J. Laws , and I. G. Jamieson . 2011. “Multimodel Inference in Ecology and Evolution: Challenges and Solutions.” Journal of Evolutionary Biology 24: 699–711. 10.1111/j.1420-9101.2010.02210.x. 21272107

[ece373417-bib-0031] Hill, G. E. , and K. J. McGraw . 2006. Bird Coloration, Volume 2: Function and Evolution. Harvard University Press.

[ece373417-bib-0032] Hylander, K. , and J. Ehrlen . 2013. “The Mechanisms Causing Extinction Debts.” Trends in Ecology & Evolution 28: 341–346. 10.1016/j.tree.2013.01.010. 23419539

[ece373417-bib-0033] Ibáñez‐Álamo, J. D. , K. Delhey , L. Izquierdo , M. Valcu , and B. Kempenaers . 2025. “Colourful Urban Birds: Bird Species Successful in Urban Environments Have More Elaborate Colours and Less Brown.” Ecology Letters 28: e70106. 10.1111/ele.70106. 40183148

[ece373417-bib-0034] Iglesias‐Carrasco, M. , D. A. Duchêne , M. L. Head , A. P. Møller , and K. Cain . 2019. “Sex in the City: Sexual Selection and Urban Colonization in Passerines.” Biology Letters 15: 20190257. 10.1098/rsbl.2019.0257. 31480935 PMC6769144

[ece373417-bib-0035] Jetz, W. , G. H. Thomas , J. B. Joy , K. Hartmann , and A. O. Mooers . 2012. “The Global Diversity of Birds in Space and Time.” Nature 491: 444–448. 10.1038/nature11631. 23123857

[ece373417-bib-0036] Jiménez, T. , I. Peña‐Villalobos , J. Arcila , F. del Basto , V. Palma , and P. Sabat . 2024. “The Effects of Urban Thermal Heterogeneity and Feather Coloration on Oxidative Stress and Metabolism of Pigeons ( *Columba livia* ).” Science of the Total Environment 912: 169564. 10.1016/j.scitotenv.2023.169564. 38142996

[ece373417-bib-0037] Johnson, M. T. , and J. Munshi‐South . 2017. “Evolution of Life in Urban Environments.” Science 358: eaam8327. 10.1126/science.aam8327. 29097520

[ece373417-bib-0038] Kamiński, M. , A. Chyb , K. D. Matson , and P. Minias . 2025. “Constitutive Innate Immune Defenses in Relation to Urbanization and Population Density in an Urban Bird, the Feral Pigeon *Columba livia* Domestica.” Integrative Zoology 20: 1136–1148. 10.1111/1749-4877.12899. 39295232

[ece373417-bib-0039] Kreling, S. E. 2023. “So Overt It's Covert: Wildlife Coloration in the City.” Bioscience 73: 333–346. 10.1093/biosci/biad021.

[ece373417-bib-0040] Leveau, L. 2021. “United Colours of the City: A Review About Urbanisation Impact on Animal Colours.” Austral Ecology 46: 670–679. 10.1111/aec.13005.

[ece373417-bib-0041] Leveau, L. M. 2019. “Urbanization Induces Bird Color Homogenization.” Landscape and Urban Planning 192: 103645. 10.1016/j.landurbplan.2019.103645.

[ece373417-bib-0042] Leveau, L. M. , and I. Ibáñez . 2022. “Nesting Site and Plumage Color Are the Main Traits Associated With Bird Species Presence in Urban Areas.” Animals 12: 1148. 10.3390/ani12091148. 35565574 PMC9099748

[ece373417-bib-0043] Leveau, L. M. , and J. Kopp . 2024. “Bird Color and Taxonomic Diversity Are Negatively Related to Human Disturbance in Urban Parks.” Web Ecology 24: 1–10. 10.5194/we-24-1-2024.

[ece373417-bib-0044] Liu, Y. , Y. Wang , J. Xie , et al. 2026. “Landscape and Habitat Effects on Functional and Phylogenetic Diversity and Structure of Bird Communities in Fragmented Habitats Within an Urban Landscape, Southwest China.” Avian Research 17: 100351. 10.1016/j.avrs.2026.100351.

[ece373417-bib-0045] Liu, Y. , Y. Zhu , S. Wu , et al. 2024. “Determinants of Taxonomic, Functional, and Phylogenetic Beta Diversity in Breeding Birds Within Urban Remnant Woodlots: Implications for Conservation.” Ecology and Evolution 14: e11426. 10.1002/ece3.11426. 38746544 PMC11091548

[ece373417-bib-0046] MacKinnon, J. R. , and K. Phillipps . 2000. A Field Guide to the Birds of China. Hunan Education Publishing House.

[ece373417-bib-0047] Novoa, R. , F. Nacaratte , I. Peña‐Villalobos , V. Palma , P. Sabat , and S. V. Copaja . 2025. “A Global Perspective on Lead in Urban Pigeons ( *Columba livia* ): Landscape, Climate, and Biological Determinants.” Journal of Hazardous Materials 497: 139524. 10.1016/j.jhazmat.2025.139524. 40834542

[ece373417-bib-0048] Roth, M. , R. A. Francis , and A. K. Hahs . 2021. The Routledge Handbook of Urban Ecology. 2nd ed. Routledge.

[ece373417-bib-0049] Roulin, A. 2014. “Melanin‐Based Colour Polymorphism Responding to Climate Change.” Global Change Biology 20: 3344–3350. 10.1111/gcb.12594. 24700793

[ece373417-bib-0050] Salmón, P. , D. López‐Idiáquez , P. Capilla‐Lasheras , J. Pérez‐Tris , C. Isaksson , and H. Watson . 2023. “Urbanisation Impacts Plumage Colouration in a Songbird Across Europe: Evidence From a Correlational, Experimental and Meta‐Analytical Approach.” Journal of Animal Ecology 92: 1924–1936. 10.1111/1365-2656.13982. 37574652

[ece373417-bib-0051] Schoereder, J. H. , C. Galbiati , C. R. Ribas , et al. 2004. “Should We Use Proportional Sampling for Species–Area Studies?” Journal of Biogeography 31: 1219–1226. 10.1111/j.1365-2699.2004.01113.x.

[ece373417-bib-0052] Shanahan, D. F. , M. W. Strohbach , P. S. Warren , and R. A. Fuller . 2013. “The Challenges of Urban Living.” In Avian Urban Ecology: Behavioural and Physiological Adaptations, edited by D. B. Gil and H. Brumm , 3–20. Oxford University Press. 10.1093/acprof:osobl/9780199661572.003.0001.

[ece373417-bib-0053] Symonds, M. R. , and A. Moussalli . 2011. “A Brief Guide to Model Selection, Multimodel Inference and Model Averaging in Behavioural Ecology Using Akaike's Information Criterion.” Behavioral Ecology and Sociobiology 65: 13–21. 10.1007/s00265-010-1037-6.

[ece373417-bib-0054] Tai, D. , C. Chen , Y. Song , X. Tan , X. Yang , and Y. Wang . 2022. “Ecological Traits and Landscape Characteristics Predicting Bird Sensitivity to Urbanization in City Parks.” Basic and Applied Ecology 58: 110–120. 10.1016/j.baae.2021.12.004.

[ece373417-bib-0055] Turak, N. , A. Monnier‐Corbel , M. Gouret , and A. Frantz . 2022. “Urbanization Shapes the Relation Between Density and Melanin‐Based Colouration in Bird Communities.” Oikos 2022: e09313. 10.1111/oik.09313.

[ece373417-bib-0056] Vincze, E. , G. Seress , M. Lagisz , S. Nakagawa , N. J. Dingemanse , and P. Sprau . 2017. “Does Urbanization Affect Predation of Bird Nests? A Meta‐Analysis.” Frontiers in Ecology and Evolution 5: 29. 10.3389/fevo.2017.00029.

[ece373417-bib-0057] Viney, C. , K. Philipps , and L. Ying . 2017. A Field Guide to Birds of Hong Kong and Southern Mainland of China. Hunan Education Publishing House.

[ece373417-bib-0058] Wang, Y. , P. Ding , S. Chen , and G. Zheng . 2013. “Nestedness of Bird Assemblages on Urban Woodlots: Implications for Conservation.” Landscape and Urban Planning 111: 59–67. 10.1016/j.landurbplan.2012.11.008.

[ece373417-bib-0059] Wu, S. , K. Zhang , B. Wang , P. Que , B. Yang , and Y. Xu . 2024. “Disentangling Ecological Drivers of Interspecific Achromatic Plumage Variation in Birds.” Global Ecology and Biogeography 33: e13892. 10.1111/geb.13892.

[ece373417-bib-0060] Xie, J. , Y. Wang , Y. Liu , et al. 2025. “Multiple Small Patches Have Higher Taxonomic, Phylogenetic, and Functional Diversity as Well as More Clustered Assemblages Than Fewer Large Patches of Comparable Area.” Landscape Ecology 40: 128. 10.1007/s10980-025-02154-5.

[ece373417-bib-0061] Yu, J. , H. Duan , B. Zhang , L. Zhang , and J. He . 2024. “Urbanization Alters the Geographic Patterns of Passerine Plumage Color in China.” Landscape and Urban Planning 248: 105101. 10.1016/j.landurbplan.2024.105101.

[ece373417-bib-0062] Zanaga, D. , R. van de Kerchove , B. van der Velde , et al. 2022. ESA WorldCover 10 m 2021 v200. Zenodo. 10.5281/zenodo.7254221.

[ece373417-bib-0063] Zheng, J. , R. Tang , S. He , et al. 2021. “Bird Diversity and Nestedness on Fragmented Woodlots in Huaxi University Town, Guizhou Province.” Biodiversity Science 29: 661–667. 10.17520/biods.2020336.

[ece373417-bib-0065] Zhu, Y. , Y. Liu , S. Sheng , et al. 2024. “Quantifying the Effects of Landscape and Habitat Characteristics on Structuring Bird Assemblages in Urban Habitat Patches.” Scientific Reports 14: 12707. 10.1038/s41598-024-63333-z. 38830929 PMC11148024

[ece373417-bib-0064] Zhu, Y. , S. Sheng , J. Zheng , S. Wu , K. Zhang , and Y. Xu . 2022. “Small‐Island Effect in Bird Assemblages on Fragmented Woodlots in Huaxi University Areas, Guizhou, China.” Chinese Journal of Zoology 57: 205–212. 10.13859/j.cjz.202202005.

