# Supplementary material for: Habitat Fragmentation in Urbanized Landscapes Favors Bird Species With Darker Plumage
Source: Ecol Evol. 2026 Apr 6;16(4):e73417. doi: 10.1002/ece3.73417 (PMC13052148; doi:10.1002/ece3.73417)
Supplement: Supplementary file 1 — Table S1: Landscape and habitat characteristics of 30 patches. Table S2: Bird species‐by‐patch presence (1)/absence (0) matrix across 30 woodlot patches. Species taxonomy and nomenclature are based on BirdTree. The Spotted Dove Stigmatopelia chinensis was excluded from the analysis due to the lack of data on achromatic plumage color. Table S3: Achromatic plumage color values of bird species recorded, shown separately for both sexes. The values represent the whole‐body mean color of each species, compiled from Delhey et al. (2021). Species taxonomy and nomenclature are based on BirdTree. The Spotted Dove Streptopelia chinensis was excluded due to the lack of data on achromatic plumage color. Table S4: Model set with cumulative Akaike weights ≤ 0.95 examining the relationships between residual achromatic plumage color (accounting for species richness) and landscape and habitat metrics for female and male birds across all, passerine, and sedentary species. Metrics include patch area, shape index (SI), the cover percentage of woodlands within patches (Woodlands), and the percentage of built‐up areas within a 500 m buffer surrounding patches (Built_up). Patch area and SI were log‐transformed, and all variables were centered and standardized. The statistics include the number of parameters (K), log‐likelihood value (logLik), Akaike's information criterion corrected for small sample size (AICc), difference in AICc relative to the minimum AICc (ΔAICc), and Akaike weights (w i ). Table S5: Model‐averaging results (from the model set with cumulative Akaike weights ≤ 0.95) for all species, and best‐supported model results for passerine and sedentary species, examining the variation in residual achromatic plumage color (accounting for species richness) in relation to landscape and habitat metrics for female and male birds. Metrics included patch area, shape index (SI), the cover percentage of woodlands within patches (Woodlands), and the percentage of built‐up areas within a 50 [file ECE3-16-e73417-s001.docx]

**Supplementary information**

**Table S1.** Landscape and habitat characteristics of 30 patches.

| Patch_No | Area  (ha) | SI | Cover percentage of woodlands within patches (%) | Percentage of built-up areas within a 500 m buffer surrounding patches (%) |
| --- | --- | --- | --- | --- |
| 1 | 2.44 | 110.91 | 95.94 | 45.30 |
| 2 | 6.19 | 160.98 | 95.92 | 49.91 |
| 3 | 9.42 | 126.00 | 90.98 | 35.30 |
| 4 | 3.56 | 116.88 | 85.38 | 42.50 |
| 5 | 2.10 | 113.56 | 83.58 | 38.38 |
| 6 | 0.92 | 135.27 | 96.64 | 41.06 |
| 7 | 0.64 | 110.83 | 82.14 | 42.07 |
| 8 | 8.65 | 194.14 | 86.52 | 50.15 |
| 9 | 2.63 | 131.61 | 90.27 | 43.61 |
| 10 | 3.69 | 156.13 | 81.46 | 48.81 |
| 11 | 0.94 | 119.27 | 73.33 | 58.48 |
| 12 | 2.29 | 128.06 | 18.58 | 55.04 |
| 13 | 1.53 | 109.46 | 95.94 | 44.08 |
| 14 | 1.49 | 177.32 | 91.84 | 30.85 |
| 15 | 3.16 | 111.69 | 14.73 | 33.70 |
| 16 | 1.16 | 112.35 | 91.39 | 35.35 |
| 17 | 290.40 | 129.78 | 67.67 | 18.45 |
| 18 | 25.50 | 201.14 | 61.60 | 31.47 |
| 19 | 6.52 | 141.84 | 85.31 | 32.39 |
| 20 | 19.03 | 146.09 | 37.20 | 30.94 |
| 21 | 34.15 | 128.57 | 33.03 | 22.93 |
| 22 | 1.38 | 112.09 | 35.00 | 25.26 |
| 23 | 1.15 | 127.00 | 25.17 | 17.90 |
| 24 | 1.40 | 132.34 | 3.89 | 24.35 |
| 25 | 11.50 | 135.47 | 88.68 | 21.32 |
| 26 | 0.25 | 110.80 | 81.82 | 35.38 |
| 27 | 114.34 | 122.17 | 35.70 | 14.17 |
| 28 | 58.36 | 180.46 | 5.90 | 20.25 |
| 29 | 1.50 | 111.33 | 91.24 | 32.23 |
| 30 | 24.21 | 110.21 | 64.23 | 18.29 |

**Table S2.** Bird species-by-patch presence (1) /absence (0) matrix across 30 woodlot patches. Species taxonomy and nomenclature are based on BirdTree. The Spotted Dove *Stigmatopelia chinensis* was excluded from the analysis due to the lack of data on achromatic plumage color.

| Species | Order | Migration | 1 | 2 | 3 | 4 | 5 | 6 | 7 | 8 | 9 | 10 | 11 | 12 | 13 | 14 | 15 | 16 | 17 | 18 | 19 | 20 | 21 | 22 | 23 | 24 | 25 | 26 | 27 | 28 | 29 | 30 |
| --- | --- | --- | --- | --- | --- | --- | --- | --- | --- | --- | --- | --- | --- | --- | --- | --- | --- | --- | --- | --- | --- | --- | --- | --- | --- | --- | --- | --- | --- | --- | --- | --- |
| Pycnonotus_xanthorrhous | Passeriformes | Sedentary | 1 | 1 | 1 | 1 | 1 | 1 | 1 | 1 | 1 | 1 | 1 | 1 | 1 | 1 | 1 | 1 | 1 | 1 | 1 | 1 | 1 | 1 | 1 | 1 | 1 | 1 | 1 | 1 | 1 | 1 |
| Garrulax_sannio | Passeriformes | Sedentary | 1 | 1 | 1 | 1 | 1 | 1 | 1 | 1 | 1 | 1 | 1 | 1 | 1 | 1 | 1 | 1 | 1 | 1 | 1 | 1 | 1 | 1 | 1 | 1 | 1 | 1 | 1 | 1 | 1 | 1 |
| Phoenicurus_auroreus | Passeriformes | Sedentary | 1 | 1 | 1 | 1 | 1 | 1 | 1 | 1 | 1 | 1 | 1 | 1 | 1 | 1 | 1 | 1 | 1 | 1 | 1 | 1 | 1 | 1 | 1 | 1 | 1 | 1 | 1 | 1 | 1 | 1 |
| Passer_montanus | Passeriformes | Sedentary | 1 | 1 | 1 | 1 | 1 | 1 | 1 | 1 | 1 | 1 | 1 | 1 | 1 | 1 | 1 | 1 | 1 | 1 | 1 | 1 | 1 | 1 | 1 | 1 | 1 | 1 | 1 | 1 | 1 | 1 |
| Motacilla_alba | Passeriformes | Sedentary | 1 | 1 | 1 | 1 | 1 | 1 | 1 | 1 | 1 | 1 | 1 | 1 | 1 | 1 | 1 | 1 | 1 | 1 | 1 | 1 | 1 | 1 | 1 | 1 | 1 | 1 | 1 | 1 | 0 | 1 |
| Spizixos_semitorques | Passeriformes | Sedentary | 1 | 1 | 1 | 1 | 1 | 1 | 1 | 1 | 1 | 1 | 1 | 1 | 1 | 1 | 1 | 1 | 1 | 1 | 1 | 1 | 1 | 1 | 1 | 1 | 1 | 1 | 1 | 1 | 1 | 1 |
| Streptopelia_orientalis | Columbiformes | Sedentary | 1 | 1 | 1 | 1 | 1 | 1 | 1 | 1 | 1 | 1 | 1 | 1 | 1 | 1 | 1 | 1 | 1 | 1 | 1 | 1 | 1 | 0 | 1 | 1 | 1 | 1 | 1 | 1 | 0 | 0 |
| Urocissa_erythrorhyncha | Passeriformes | Sedentary | 1 | 1 | 1 | 1 | 1 | 1 | 1 | 1 | 1 | 1 | 1 | 1 | 1 | 1 | 1 | 1 | 1 | 1 | 1 | 1 | 1 | 1 | 1 | 0 | 1 | 1 | 1 | 1 | 1 | 0 |
| Parus_major | Passeriformes | Sedentary | 1 | 1 | 1 | 1 | 1 | 1 | 1 | 1 | 1 | 1 | 0 | 1 | 1 | 1 | 1 | 1 | 1 | 1 | 1 | 1 | 1 | 1 | 1 | 1 | 1 | 1 | 1 | 1 | 1 | 1 |
| Carduelis_sinica | Passeriformes | Sedentary | 1 | 1 | 1 | 1 | 1 | 1 | 1 | 1 | 1 | 1 | 1 | 1 | 1 | 1 | 1 | 0 | 1 | 1 | 1 | 1 | 1 | 1 | 1 | 1 | 1 | 1 | 1 | 1 | 1 | 1 |
| Pica_pica | Passeriformes | Sedentary | 0 | 1 | 1 | 1 | 1 | 1 | 1 | 1 | 1 | 1 | 1 | 1 | 1 | 1 | 1 | 0 | 1 | 1 | 1 | 1 | 1 | 1 | 1 | 1 | 1 | 1 | 1 | 1 | 1 | 1 |
| Lanius_schach | Passeriformes | Sedentary | 0 | 1 | 1 | 1 | 0 | 1 | 1 | 1 | 1 | 1 | 1 | 1 | 1 | 1 | 1 | 1 | 1 | 1 | 0 | 1 | 1 | 1 | 1 | 1 | 1 | 1 | 1 | 1 | 0 | 1 |
| Cettia_fortipes | Passeriformes | Sedentary | 1 | 1 | 1 | 1 | 1 | 0 | 0 | 1 | 1 | 1 | 0 | 1 | 1 | 0 | 1 | 1 | 1 | 1 | 0 | 1 | 1 | 1 | 1 | 1 | 1 | 0 | 1 | 1 | 0 | 1 |
| Copsychus_saularis | Passeriformes | Sedentary | 1 | 1 | 1 | 1 | 1 | 1 | 1 | 1 | 1 | 1 | 0 | 1 | 1 | 1 | 1 | 1 | 1 | 1 | 1 | 1 | 1 | 0 | 0 | 0 | 1 | 1 | 1 | 1 | 0 | 1 |
| Zosterops_japonicus | Passeriformes | Summer migrant | 1 | 1 | 1 | 1 | 1 | 1 | 0 | 1 | 1 | 0 | 0 | 1 | 1 | 1 | 0 | 1 | 1 | 1 | 1 | 1 | 1 | 1 | 0 | 0 | 1 | 1 | 1 | 1 | 1 | 1 |
| Aegithalos_concinnus | Passeriformes | Sedentary | 1 | 1 | 1 | 0 | 0 | 1 | 0 | 1 | 1 | 1 | 0 | 0 | 1 | 1 | 0 | 1 | 1 | 1 | 0 | 1 | 1 | 0 | 0 | 0 | 1 | 0 | 1 | 1 | 0 | 1 |
| Acridotheres_cristatellus | Passeriformes | Sedentary | 0 | 0 | 1 | 0 | 0 | 0 | 0 | 1 | 0 | 0 | 0 | 1 | 0 | 0 | 1 | 1 | 1 | 1 | 1 | 1 | 1 | 0 | 1 | 1 | 1 | 0 | 1 | 1 | 1 | 1 |
| Phylloscopus_proregulus | Passeriformes | Summer migrant | 1 | 1 | 1 | 1 | 1 | 1 | 1 | 1 | 1 | 0 | 0 | 0 | 0 | 1 | 0 | 0 | 1 | 0 | 0 | 1 | 0 | 0 | 0 | 0 | 1 | 0 | 1 | 1 | 1 | 0 |
| Abroscopus_albogularis | Passeriformes | Sedentary | 1 | 1 | 1 | 1 | 0 | 0 | 0 | 1 | 1 | 1 | 0 | 0 | 0 | 1 | 0 | 1 | 1 | 1 | 1 | 1 | 1 | 0 | 0 | 0 | 1 | 0 | 1 | 0 | 0 | 1 |
| Prinia_inornata | Passeriformes | Sedentary | 0 | 0 | 0 | 0 | 0 | 0 | 0 | 0 | 0 | 0 | 1 | 1 | 0 | 0 | 1 | 0 | 1 | 1 | 0 | 1 | 1 | 1 | 1 | 1 | 0 | 0 | 1 | 1 | 0 | 0 |
| Lanius_tigrinus | Passeriformes | Summer migrant | 0 | 0 | 0 | 0 | 0 | 1 | 0 | 1 | 1 | 0 | 0 | 0 | 0 | 1 | 0 | 0 | 1 | 0 | 1 | 0 | 0 | 0 | 0 | 1 | 1 | 0 | 1 | 1 | 0 | 1 |
| Turdus_merula | Passeriformes | Sedentary | 0 | 0 | 1 | 0 | 0 | 0 | 1 | 1 | 1 | 0 | 1 | 1 | 0 | 1 | 0 | 1 | 1 | 1 | 1 | 1 | 0 | 0 | 0 | 0 | 1 | 1 | 1 | 1 | 0 | 0 |
| Stigmatopelia_chinensis | Columbiformes | Sedentary | 1 | 1 | 1 | 1 | 0 | 0 | 0 | 0 | 0 | 0 | 0 | 1 | 1 | 1 | 1 | 0 | 1 | 1 | 1 | 1 | 1 | 0 | 0 | 1 | 1 | 0 | 1 | 1 | 1 | 0 |
| Lanius_cristatus | Passeriformes | Summer migrant | 1 | 0 | 0 | 0 | 0 | 0 | 0 | 1 | 0 | 0 | 0 | 0 | 1 | 1 | 0 | 0 | 1 | 0 | 0 | 1 | 1 | 0 | 0 | 1 | 1 | 0 | 1 | 1 | 0 | 0 |
| Saxicola_torquatus | Passeriformes | Sedentary | 0 | 0 | 0 | 0 | 0 | 0 | 0 | 1 | 0 | 0 | 0 | 0 | 0 | 0 | 1 | 0 | 1 | 0 | 0 | 1 | 1 | 1 | 1 | 1 | 0 | 0 | 0 | 1 | 0 | 0 |
| Lonchura_striata | Passeriformes | Sedentary | 0 | 0 | 0 | 0 | 0 | 0 | 0 | 0 | 1 | 0 | 0 | 0 | 0 | 0 | 0 | 0 | 1 | 0 | 1 | 1 | 1 | 1 | 0 | 1 | 0 | 0 | 0 | 0 | 0 | 0 |
| Eophona_migratoria | Passeriformes | Summer migrant | 0 | 0 | 1 | 0 | 0 | 0 | 0 | 1 | 1 | 0 | 0 | 0 | 0 | 0 | 0 | 1 | 1 | 1 | 1 | 0 | 0 | 0 | 0 | 0 | 1 | 1 | 0 | 0 | 0 | 0 |
| Egretta_garzetta | Pelecaniformes | Sedentary | 0 | 0 | 0 | 0 | 0 | 0 | 0 | 0 | 1 | 0 | 0 | 0 | 0 | 1 | 0 | 1 | 1 | 1 | 0 | 1 | 1 | 0 | 0 | 0 | 1 | 0 | 1 | 0 | 0 | 0 |
| Eudynamys_scolopaceus | Cuculiformes | Summer migrant | 1 | 0 | 1 | 0 | 0 | 0 | 0 | 0 | 1 | 0 | 0 | 1 | 1 | 1 | 0 | 0 | 1 | 1 | 1 | 1 | 1 | 0 | 0 | 0 | 1 | 1 | 1 | 1 | 0 | 1 |
| Pericrocotus_roseus | Passeriformes | Summer migrant | 0 | 1 | 0 | 0 | 0 | 0 | 0 | 1 | 0 | 1 | 1 | 0 | 0 | 0 | 0 | 0 | 1 | 0 | 1 | 0 | 0 | 0 | 0 | 0 | 1 | 0 | 1 | 0 | 0 | 0 |
| Turdus_dissimilis | Passeriformes | Sedentary | 0 | 0 | 0 | 0 | 0 | 0 | 0 | 1 | 1 | 1 | 0 | 1 | 1 | 1 | 0 | 1 | 1 | 1 | 1 | 1 | 0 | 0 | 0 | 0 | 1 | 1 | 1 | 1 | 0 | 0 |
| Monticola_solitarius | Passeriformes | Sedentary | 1 | 0 | 1 | 0 | 0 | 0 | 0 | 1 | 0 | 0 | 0 | 1 | 0 | 0 | 0 | 0 | 0 | 1 | 1 | 0 | 0 | 0 | 1 | 0 | 1 | 0 | 0 | 1 | 0 | 0 |
| Myophonus_caeruleus | Passeriformes | Sedentary | 0 | 0 | 1 | 0 | 0 | 0 | 0 | 1 | 0 | 0 | 1 | 0 | 0 | 0 | 0 | 0 | 1 | 1 | 0 | 1 | 1 | 0 | 0 | 0 | 1 | 0 | 0 | 1 | 0 | 0 |
| Alcippe_dubia | Passeriformes | Sedentary | 1 | 1 | 1 | 1 | 0 | 1 | 1 | 1 | 1 | 1 | 1 | 0 | 0 | 0 | 0 | 1 | 1 | 1 | 0 | 1 | 1 | 0 | 0 | 0 | 1 | 0 | 1 | 1 | 1 | 1 |
| Passer_rutilans | Passeriformes | Sedentary | 0 | 0 | 0 | 0 | 0 | 0 | 0 | 0 | 1 | 1 | 0 | 0 | 0 | 1 | 0 | 1 | 1 | 0 | 1 | 0 | 0 | 0 | 0 | 0 | 1 | 0 | 1 | 0 | 0 | 0 |
| Motacilla_cinerea | Passeriformes | Sedentary | 0 | 0 | 0 | 0 | 0 | 0 | 0 | 0 | 0 | 0 | 0 | 0 | 0 | 0 | 0 | 1 | 1 | 1 | 1 | 1 | 1 | 0 | 0 | 0 | 0 | 0 | 0 | 0 | 0 | 0 |
| Rhyacornis_fuliginosa | Passeriformes | Sedentary | 0 | 0 | 0 | 0 | 0 | 0 | 0 | 0 | 0 | 0 | 0 | 0 | 0 | 0 | 0 | 0 | 1 | 1 | 1 | 1 | 1 | 0 | 0 | 0 | 1 | 0 | 0 | 0 | 0 | 0 |
| Pomatorhinus_gravivox | Passeriformes | Sedentary | 0 | 0 | 0 | 0 | 0 | 0 | 0 | 0 | 0 | 0 | 0 | 0 | 0 | 1 | 0 | 1 | 1 | 1 | 0 | 1 | 1 | 0 | 0 | 1 | 0 | 0 | 1 | 1 | 0 | 1 |
| Paradoxornis_alphonsianus | Passeriformes | Sedentary | 1 | 0 | 1 | 0 | 0 | 0 | 0 | 0 | 0 | 0 | 0 | 0 | 0 | 1 | 0 | 0 | 1 | 1 | 1 | 0 | 1 | 0 | 0 | 0 | 1 | 0 | 1 | 1 | 0 | 1 |
| Prinia_crinigera | Passeriformes | Sedentary | 0 | 0 | 0 | 0 | 0 | 0 | 0 | 0 | 0 | 0 | 1 | 0 | 0 | 0 | 1 | 0 | 1 | 1 | 0 | 1 | 1 | 1 | 1 | 0 | 0 | 0 | 1 | 1 | 0 | 1 |
| Ardeola_bacchus | Pelecaniformes | Summer migrant | 0 | 0 | 0 | 0 | 0 | 0 | 0 | 0 | 0 | 0 | 0 | 0 | 0 | 0 | 0 | 1 | 1 | 1 | 0 | 1 | 1 | 0 | 0 | 0 | 1 | 0 | 0 | 0 | 0 | 1 |
| Lonchura_punctulata | Passeriformes | Sedentary | 0 | 0 | 0 | 0 | 0 | 0 | 1 | 0 | 0 | 1 | 0 | 1 | 0 | 0 | 1 | 0 | 1 | 1 | 0 | 1 | 1 | 0 | 0 | 0 | 0 | 0 | 0 | 1 | 0 | 0 |
| Stachyris_ruficeps | Passeriformes | Sedentary | 1 | 1 | 1 | 0 | 0 | 1 | 0 | 0 | 0 | 0 | 0 | 0 | 0 | 1 | 0 | 1 | 1 | 1 | 0 | 1 | 1 | 0 | 0 | 0 | 1 | 1 | 1 | 0 | 0 | 1 |
| Sturnus_sericeus | Passeriformes | Sedentary | 0 | 0 | 0 | 0 | 0 | 0 | 1 | 0 | 0 | 0 | 0 | 0 | 0 | 0 | 0 | 1 | 1 | 0 | 1 | 1 | 0 | 0 | 0 | 0 | 1 | 0 | 1 | 0 | 0 | 1 |
| Terpsiphone_paradisi | Passeriformes | Summer migrant | 1 | 0 | 0 | 0 | 0 | 0 | 0 | 0 | 0 | 0 | 0 | 0 | 0 | 0 | 0 | 0 | 0 | 0 | 1 | 0 | 0 | 0 | 0 | 0 | 0 | 0 | 0 | 0 | 0 | 0 |
| Pomatorhinus_ruficollis | Passeriformes | Sedentary | 0 | 0 | 1 | 0 | 0 | 0 | 0 | 0 | 1 | 0 | 0 | 0 | 0 | 0 | 0 | 1 | 1 | 1 | 0 | 0 | 1 | 0 | 0 | 0 | 0 | 0 | 1 | 0 | 0 | 1 |
| Emberiza_elegans | Passeriformes | Sedentary | 0 | 0 | 0 | 0 | 0 | 0 | 0 | 0 | 0 | 0 | 0 | 0 | 0 | 0 | 0 | 0 | 1 | 1 | 0 | 1 | 0 | 0 | 0 | 0 | 0 | 0 | 1 | 1 | 0 | 1 |
| Dicrurus_hottentottus | Passeriformes | Summer migrant | 0 | 1 | 0 | 0 | 0 | 0 | 0 | 0 | 0 | 0 | 0 | 0 | 0 | 0 | 0 | 0 | 0 | 1 | 1 | 0 | 0 | 0 | 0 | 0 | 1 | 0 | 1 | 0 | 0 | 0 |
| Garrulus_glandarius | Passeriformes | Sedentary | 0 | 0 | 0 | 0 | 0 | 0 | 0 | 0 | 0 | 0 | 0 | 0 | 1 | 1 | 0 | 0 | 1 | 0 | 0 | 0 | 0 | 0 | 0 | 0 | 0 | 1 | 1 | 0 | 0 | 1 |
| Eumyias_thalassinus | Passeriformes | Summer migrant | 1 | 0 | 0 | 0 | 0 | 0 | 0 | 0 | 1 | 0 | 0 | 0 | 0 | 1 | 0 | 0 | 1 | 1 | 1 | 0 | 0 | 0 | 0 | 1 | 1 | 0 | 1 | 0 | 0 | 0 |
| Muscicapa_dauurica | Passeriformes | Summer migrant | 0 | 0 | 0 | 0 | 0 | 0 | 0 | 0 | 0 | 0 | 0 | 0 | 1 | 0 | 0 | 0 | 0 | 0 | 0 | 1 | 0 | 0 | 0 | 0 | 1 | 0 | 0 | 1 | 0 | 1 |
| Yuhina_castaniceps | Passeriformes | Sedentary | 0 | 0 | 1 | 0 | 0 | 0 | 0 | 0 | 0 | 1 | 0 | 0 | 0 | 0 | 0 | 0 | 1 | 0 | 0 | 0 | 0 | 0 | 0 | 0 | 0 | 0 | 0 | 0 | 0 | 1 |
| Emberiza_godlewskii | Passeriformes | Sedentary | 0 | 0 | 1 | 0 | 0 | 0 | 0 | 0 | 0 | 0 | 0 | 0 | 0 | 0 | 0 | 0 | 1 | 0 | 0 | 0 | 0 | 0 | 0 | 0 | 0 | 0 | 1 | 1 | 0 | 1 |
| Amaurornis_akool | Gruiformes | Summer migrant | 0 | 0 | 0 | 0 | 0 | 0 | 0 | 0 | 0 | 0 | 0 | 0 | 0 | 0 | 0 | 0 | 0 | 0 | 0 | 1 | 1 | 0 | 0 | 0 | 0 | 0 | 0 | 0 | 0 | 0 |
| Phasianus_colchicus | Galliformes | Sedentary | 0 | 0 | 0 | 0 | 0 | 0 | 0 | 0 | 0 | 0 | 0 | 0 | 0 | 0 | 1 | 0 | 0 | 0 | 0 | 1 | 0 | 0 | 0 | 0 | 0 | 0 | 1 | 1 | 0 | 0 |
| Emberiza_fucata | Passeriformes | Summer migrant | 0 | 0 | 0 | 0 | 0 | 0 | 0 | 0 | 0 | 0 | 0 | 0 | 0 | 0 | 1 | 0 | 1 | 0 | 0 | 0 | 0 | 0 | 0 | 0 | 0 | 0 | 0 | 0 | 0 | 0 |
| Tachybaptus_ruficollis | Podicipediformes | Sedentary | 0 | 0 | 0 | 0 | 0 | 0 | 0 | 0 | 0 | 0 | 0 | 0 | 0 | 0 | 0 | 0 | 1 | 0 | 0 | 0 | 0 | 0 | 0 | 0 | 0 | 0 | 0 | 0 | 0 | 0 |
| Alcedo_atthis | Coraciiformes | Sedentary | 0 | 0 | 0 | 0 | 0 | 0 | 0 | 0 | 0 | 0 | 1 | 0 | 0 | 0 | 0 | 0 | 0 | 0 | 1 | 0 | 1 | 0 | 0 | 0 | 1 | 0 | 0 | 0 | 0 | 0 |
| Megalaima_virens | Piciformes | Sedentary | 0 | 0 | 0 | 0 | 0 | 0 | 0 | 0 | 0 | 0 | 0 | 0 | 0 | 0 | 0 | 0 | 0 | 0 | 0 | 0 | 0 | 0 | 0 | 0 | 1 | 0 | 1 | 0 | 0 | 0 |
| Picus_canus | Piciformes | Sedentary | 0 | 0 | 0 | 0 | 0 | 0 | 0 | 0 | 0 | 0 | 0 | 0 | 0 | 0 | 0 | 0 | 0 | 0 | 0 | 0 | 0 | 0 | 0 | 0 | 1 | 0 | 1 | 0 | 0 | 0 |
| Pericrocotus_cantonensis | Passeriformes | Summer migrant | 0 | 0 | 0 | 0 | 0 | 0 | 0 | 1 | 0 | 0 | 0 | 0 | 0 | 0 | 0 | 0 | 0 | 0 | 1 | 0 | 0 | 0 | 0 | 0 | 0 | 0 | 1 | 0 | 0 | 1 |
| Chaimarrornis_leucocephalus | Passeriformes | Sedentary | 0 | 0 | 0 | 1 | 0 | 0 | 0 | 0 | 0 | 0 | 0 | 0 | 0 | 0 | 0 | 0 | 0 | 1 | 0 | 0 | 0 | 0 | 0 | 0 | 0 | 0 | 0 | 0 | 0 | 0 |
| Emberiza_cioides | Passeriformes | Sedentary | 0 | 0 | 0 | 0 | 0 | 0 | 0 | 0 | 0 | 0 | 0 | 0 | 0 | 0 | 0 | 0 | 1 | 1 | 0 | 0 | 0 | 0 | 0 | 0 | 0 | 0 | 0 | 0 | 0 | 0 |
| Culicicapa_ceylonensis | Passeriformes | Summer migrant | 0 | 1 | 0 | 0 | 0 | 0 | 0 | 0 | 0 | 0 | 0 | 0 | 0 | 0 | 0 | 0 | 0 | 0 | 0 | 0 | 0 | 0 | 0 | 0 | 1 | 0 | 0 | 1 | 0 | 0 |
| Phylloscopus_trochiloides | Passeriformes | Summer migrant | 0 | 0 | 0 | 0 | 0 | 0 | 0 | 0 | 1 | 0 | 0 | 0 | 0 | 0 | 0 | 0 | 0 | 0 | 0 | 0 | 0 | 0 | 0 | 0 | 1 | 0 | 0 | 0 | 0 | 0 |
| Pycnonotus_sinensis | Passeriformes | Sedentary | 0 | 0 | 0 | 0 | 0 | 0 | 0 | 0 | 0 | 0 | 0 | 0 | 0 | 0 | 0 | 0 | 0 | 0 | 0 | 0 | 1 | 0 | 0 | 1 | 0 | 0 | 0 | 0 | 0 | 0 |
| Hypsipetes_mcclellandii | Passeriformes | Sedentary | 0 | 1 | 0 | 0 | 0 | 0 | 0 | 0 | 0 | 0 | 0 | 0 | 0 | 0 | 0 | 0 | 0 | 0 | 0 | 0 | 0 | 0 | 1 | 0 | 0 | 0 | 0 | 0 | 0 | 1 |
| Cuculus_canorus | Cuculiformes | Summer migrant | 0 | 0 | 0 | 0 | 0 | 0 | 0 | 0 | 0 | 0 | 0 | 0 | 0 | 0 | 0 | 0 | 1 | 0 | 0 | 0 | 0 | 0 | 0 | 0 | 0 | 0 | 1 | 1 | 0 | 0 |
| Saxicola_ferreus | Passeriformes | Sedentary | 0 | 0 | 0 | 0 | 0 | 0 | 0 | 0 | 0 | 0 | 0 | 0 | 0 | 0 | 0 | 0 | 0 | 0 | 0 | 0 | 0 | 0 | 0 | 0 | 0 | 0 | 1 | 1 | 0 | 1 |
| Dicrurus_leucophaeus | Passeriformes | Summer migrant | 0 | 0 | 0 | 0 | 0 | 0 | 0 | 0 | 0 | 0 | 0 | 0 | 0 | 0 | 0 | 0 | 1 | 0 | 0 | 0 | 0 | 0 | 0 | 0 | 1 | 0 | 1 | 0 | 0 | 0 |
| Acrocephalus_concinens | Passeriformes | Summer migrant | 0 | 0 | 0 | 0 | 0 | 0 | 0 | 0 | 0 | 0 | 0 | 0 | 0 | 0 | 0 | 0 | 0 | 0 | 0 | 0 | 0 | 0 | 0 | 0 | 0 | 0 | 1 | 0 | 0 | 1 |
| Paradoxornis_webbianus | Passeriformes | Sedentary | 1 | 0 | 0 | 0 | 0 | 0 | 0 | 0 | 0 | 0 | 0 | 0 | 0 | 0 | 0 | 0 | 1 | 1 | 0 | 0 | 1 | 0 | 0 | 0 | 0 | 0 | 1 | 0 | 0 | 1 |
| Cuculus_sparverioides | Cuculiformes | Summer migrant | 0 | 0 | 0 | 0 | 0 | 0 | 0 | 0 | 0 | 1 | 0 | 0 | 0 | 0 | 0 | 0 | 1 | 0 | 1 | 0 | 0 | 0 | 0 | 0 | 0 | 0 | 1 | 1 | 0 | 0 |
| Hypsipetes_leucocephalus | Passeriformes | Sedentary | 0 | 0 | 0 | 0 | 0 | 0 | 0 | 0 | 1 | 0 | 0 | 0 | 0 | 0 | 0 | 0 | 0 | 0 | 0 | 0 | 0 | 0 | 0 | 0 | 0 | 0 | 0 | 0 | 0 | 1 |
| Cyornis_banyumas | Passeriformes | Summer migrant | 0 | 0 | 0 | 0 | 0 | 0 | 0 | 0 | 0 | 0 | 0 | 0 | 1 | 0 | 0 | 0 | 0 | 0 | 0 | 0 | 0 | 0 | 0 | 0 | 0 | 0 | 0 | 0 | 0 | 0 |
| Picumnus_innominatus | Piciformes | Sedentary | 0 | 0 | 1 | 0 | 0 | 0 | 0 | 1 | 0 | 0 | 0 | 0 | 0 | 1 | 0 | 0 | 1 | 0 | 0 | 1 | 0 | 0 | 0 | 0 | 0 | 0 | 0 | 0 | 0 | 1 |
| Dicrurus_macrocercus | Passeriformes | Summer migrant | 0 | 0 | 0 | 0 | 0 | 0 | 0 | 0 | 0 | 0 | 0 | 0 | 0 | 1 | 0 | 0 | 1 | 0 | 0 | 0 | 0 | 0 | 0 | 0 | 0 | 0 | 0 | 0 | 0 | 1 |
| Leiothrix_lutea | Passeriformes | Sedentary | 0 | 0 | 0 | 0 | 0 | 0 | 0 | 0 | 0 | 0 | 0 | 0 | 0 | 0 | 0 | 0 | 1 | 0 | 0 | 0 | 0 | 0 | 0 | 0 | 0 | 0 | 0 | 0 | 0 | 1 |
| Ardea_cinerea | Pelecaniformes | Sedentary | 0 | 0 | 1 | 0 | 0 | 0 | 0 | 0 | 0 | 0 | 0 | 0 | 0 | 0 | 0 | 0 | 1 | 1 | 1 | 0 | 1 | 0 | 0 | 0 | 1 | 0 | 0 | 0 | 0 | 1 |
| Zosterops_palpebrosus | Passeriformes | Sedentary | 0 | 0 | 1 | 0 | 0 | 0 | 0 | 0 | 0 | 0 | 0 | 0 | 0 | 0 | 0 | 0 | 1 | 0 | 0 | 0 | 0 | 0 | 0 | 0 | 0 | 0 | 0 | 0 | 0 | 0 |
| Bambusicola_thoracicus | Galliformes | Sedentary | 0 | 0 | 0 | 0 | 0 | 0 | 0 | 0 | 0 | 0 | 0 | 0 | 0 | 0 | 0 | 0 | 1 | 0 | 0 | 0 | 0 | 0 | 0 | 0 | 0 | 0 | 1 | 0 | 0 | 0 |
| Nycticorax_nycticorax | Pelecaniformes | Summer migrant | 0 | 0 | 0 | 0 | 0 | 0 | 0 | 0 | 0 | 0 | 0 | 0 | 0 | 0 | 0 | 0 | 0 | 1 | 0 | 1 | 0 | 0 | 0 | 0 | 0 | 0 | 0 | 0 | 0 | 0 |

**Table S3.** Achromatic plumage color values of bird species recorded, shown separately for both sexes. The values represent the whole-body mean color of each species, compiled from Delhey et al. (2021). Species taxonomy and nomenclature are based on BirdTree. The Spotted Dove *Streptopelia chinensis* was excluded due to the lack of data on achromatic plumage color.

| Species | Order | Female | Male |
| --- | --- | --- | --- |
| Pycnonotus_xanthorrhous | Passeriformes | 63.92 | 63.92 |
| Garrulax_sannio | Passeriformes | 57.89 | 57.89 |
| Phoenicurus_auroreus | Passeriformes | 58.72 | 52.27 |
| Passer_montanus | Passeriformes | 66.69 | 66.69 |
| Motacilla_alba | Passeriformes | 62.62 | 61.65 |
| Spizixos_semitorques | Passeriformes | 60.41 | 60.41 |
| Streptopelia_orientalis | Columbiformes | 55.13 | 55.13 |
| Urocissa_erythrorhyncha | Passeriformes | 66.81 | 66.81 |
| Parus_major | Passeriformes | 69.02 | 66.09 |
| Carduelis_sinica | Passeriformes | 70.13 | 66.40 |
| Pica_pica | Passeriformes | 49.16 | 49.16 |
| Lanius_schach | Passeriformes | 63.49 | 63.49 |
| Cettia_fortipes | Passeriformes | 58.27 | 58.27 |
| Copsychus_saularis | Passeriformes | 62.42 | 55.36 |
| Zosterops_japonicus | Passeriformes | 59.25 | 59.25 |
| Aegithalos_concinnus | Passeriformes | 62.77 | 62.77 |
| Acridotheres_cristatellus | Passeriformes | 44.88 | 44.88 |
| Phylloscopus_proregulus | Passeriformes | 58.61 | 58.61 |
| Abroscopus_albogularis | Passeriformes | 63.26 | 63.26 |
| Prinia_inornata | Passeriformes | 74.78 | 74.78 |
| Lanius_tigrinus | Passeriformes | 73.15 | 67.61 |
| Turdus_merula | Passeriformes | 47.85 | 40.82 |
| Lanius_cristatus | Passeriformes | 71.19 | 69.69 |
| Saxicola_torquatus | Passeriformes | 56.33 | 54.42 |
| Lonchura_striata | Passeriformes | 57.53 | 57.53 |
| Eophona_migratoria | Passeriformes | 71.09 | 68.44 |
| Egretta_garzetta | Pelecaniformes | 82.62 | 82.62 |
| Eudynamys_scolopaceus | Cuculiformes | 49.82 | 34.58 |
| Pericrocotus_roseus | Passeriformes | 62.31 | 56.48 |
| Turdus_dissimilis | Passeriformes | 55.80 | 50.77 |
| Monticola_solitarius | Passeriformes | 52.95 | 51.08 |
| Myophonus_caeruleus | Passeriformes | 44.84 | 44.84 |
| Alcippe_dubia | Passeriformes | 61.54 | 61.54 |
| Passer_rutilans | Passeriformes | 69.59 | 67.24 |
| Motacilla_cinerea | Passeriformes | 62.06 | 59.58 |
| Rhyacornis_fuliginosa | Passeriformes | 57.53 | 41.01 |
| Pomatorhinus_gravivox | Passeriformes | 54.10 | 54.10 |
| Paradoxornis_alphonsianus | Passeriformes | 53.82 | 53.82 |
| Prinia_crinigera | Passeriformes | 64.47 | 64.47 |
| Ardeola_bacchus | Pelecaniformes | 66.86 | 66.86 |
| Lonchura_punctulata | Passeriformes | 62.47 | 62.47 |
| Stachyris_ruficeps | Passeriformes | 54.56 | 54.56 |
| Sturnus_sericeus | Passeriformes | 67.26 | 72.62 |
| Terpsiphone_paradisi | Passeriformes | 66.20 | 74.57 |
| Pomatorhinus_ruficollis | Passeriformes | 57.55 | 57.55 |
| Emberiza_elegans | Passeriformes | 71.05 | 69.71 |
| Dicrurus_hottentottus | Passeriformes | 50.48 | 50.48 |
| Garrulus_glandarius | Passeriformes | 61.39 | 61.39 |
| Eumyias_thalassinus | Passeriformes | 66.93 | 67.36 |
| Muscicapa_dauurica | Passeriformes | 60.48 | 60.48 |
| Yuhina_castaniceps | Passeriformes | 70.25 | 70.25 |
| Emberiza_godlewskii | Passeriformes | 63.52 | 59.28 |
| Amaurornis_akool | Gruiformes | 58.51 | 58.51 |
| Phasianus_colchicus | Galliformes | 58.78 | 56.87 |
| Emberiza_fucata | Passeriformes | 66.16 | 64.02 |
| Tachybaptus_ruficollis | Podicipediformes | 39.51 | 39.51 |
| Alcedo_atthis | Coraciiformes | 58.30 | 53.03 |
| Megalaima_virens | Piciformes | 53.76 | 53.76 |
| Picus_canus | Piciformes | 54.85 | 54.94 |
| Pericrocotus_cantonensis | Passeriformes | 62.21 | 63.22 |
| Chaimarrornis_leucocephalus | Passeriformes | 44.68 | 44.68 |
| Emberiza_cioides | Passeriformes | 62.97 | 59.71 |
| Culicicapa_ceylonensis | Passeriformes | 62.88 | 62.88 |
| Phylloscopus_trochiloides | Passeriformes | 67.90 | 67.90 |
| Pycnonotus_sinensis | Passeriformes | 66.26 | 66.26 |
| Hypsipetes_mcclellandii | Passeriformes | 56.35 | 56.35 |
| Cuculus_canorus | Cuculiformes | 55.14 | 53.83 |
| Saxicola_ferreus | Passeriformes | 57.70 | 61.90 |
| Dicrurus_leucophaeus | Passeriformes | 60.34 | 60.34 |
| Acrocephalus_concinens | Passeriformes | 68.32 | 68.32 |
| Paradoxornis_webbianus | Passeriformes | 60.27 | 60.27 |
| Cuculus_sparverioides | Cuculiformes | 50.44 | 50.44 |
| Hypsipetes_leucocephalus | Passeriformes | 46.41 | 46.41 |
| Cyornis_banyumas | Passeriformes | 61.16 | 52.29 |
| Picumnus_innominatus | Piciformes | 58.10 | 59.66 |
| Dicrurus_macrocercus | Passeriformes | 49.73 | 49.73 |
| Leiothrix_lutea | Passeriformes | 59.44 | 59.44 |
| Ardea_cinerea | Pelecaniformes | 70.06 | 70.06 |
| Zosterops_palpebrosus | Passeriformes | 67.24 | 67.24 |
| Bambusicola_thoracicus | Galliformes | 70.28 | 70.28 |
| Nycticorax_nycticorax | Pelecaniformes | 63.81 | 63.81 |

**Table S4**. Model set with cumulative Akaike weights ≤ 0.95 examining the relationships between residual achromatic plumage color (accounting for species richness) and landscape and habitat metrics for female and male birds across all, passerine, and sedentary species. Metrics include patch area, shape index (SI), the cover percentage of woodlands within patches (Woodlands), and the percentage of built-up areas within a 500 m buffer surrounding patches (Built_up). Patch area and SI were log-transformed, and all variables were centered and standardized. The statistics include the number of parameters (*K*), log-likelihood value (*logLik*), Akaike’s information criterion corrected for small sample size (*AICc*), difference in *AICc* relative to the minimum *AICc* (Δ*AICc*), and Akaike weights (*w_i_*).

| Group | Model | *K* | *logLik* | *AICc* | | Δ*AICc* | *w_i_* |
| --- | --- | --- | --- | --- | --- | --- | --- |
| *All species* | *Female* |  |  |  | |  |  |
|  | Patch area + SI | 4 | -186.83 | 383.26 | | 0 | 0.28 |
|  | SI + Built_up | 4 | -187.57 | 384.75 | | 1.49 | 0.13 |
|  | Patch area +SI + Built_up | 5 | -186.26 | 385.03 | | 1.77 | 0.12 |
|  | Built_up | 3 | -189.13 | 385.19 | | 1.93 | 0.11 |
|  | Patch area + SI + Woodlands | 5 | -186.79 | 386.09 | | 2.83 | 0.07 |
|  | SI | 3 | -190.00 | 386.92 | | 3.66 | 0.04 |
|  | Built_up+SI +Woodlands | 5 | -187.36 | 387.22 | | 3.96 | 0.04 |
|  | Built_up+ Woodlands | 4 | -188.85 | 387.30 | | 4.04 | 0.04 |
|  | Null | 2 | -191.43 | 387.31 | | 4.05 | 0.04 |
|  | Patch area | 3 | -190.25 | 387.42 | | 4.16 | 0.03 |
|  | Patch area +Built_up | 4 | -188.96 | 387.53 | | 4.27 | 0.03 |
|  | *Male* | | | |  |  |  |
|  | Patch area + SI | 4 | -186.33 | 382.26 | | 0 | 0.30 |
|  | SI + Built_up | 4 | -187.09 | 383.79 | | 1.53 | 0.14 |
|  | Patch area + SI + Built_up | 5 | -185.66 | 383.83 | | 1.57 | 0.14 |
|  | Built_up | 3 | -188.81 | 384.55 | | 2.29 | 0.09 |
|  | Patch area + SI + Woodlands | 5 | -186.31 | 385.12 | | 2.86 | 0.07 |
|  | Built_up + SI + Woodlands | 5 | -186.90 | 386.30 | | 4.05 | 0.04 |
|  | Patch area + Built_up + SI + Woodlands | 6 | -185.42 | 386.49 | | 4.24 | 0.04 |
|  | SI | 3 | -189.81 | 386.55 | | 4.29 | 0.03 |
|  | Built_up + Woodlands | 4 | -188.56 | 386.71 | | 4.45 | 0.03 |
|  | Patch area + Built_up | 4 | -188.64 | 386.87 | | 4.61 | 0.03 |
|  | Patch area | 3 | -190.08 | 387.09 | | 4.83 | 0.03 |
| *Passerine species* | *Female* | | | | | | |
|  | Patch area + SI | 4 | -175.92 | 361.44 | | 0 | 0.37 |
|  | Patch area +SI + Built_up | 5 | -175.71 | 363.92 | | 2.48 | 0.11 |
|  | Patch area +SI + Woodlands | 5 | -175.86 | 364.22 | | 2.78 | 0.09 |
|  | Built_up +SI | 4 | -177.37 | 364.34 | | 2.90 | 0.09 |
|  | Built_up | 3 | -179.02 | 364.96 | | 3.52 | 0.06 |
|  | SI | 3 | -179.12 | 365.15 | | 3.71 | 0.06 |
|  | Null | 2 | -180.68 | 365.80 | | 4.36 | 0.04 |
|  | Patch area | 3 | -179.54 | 366.00 | | 4.56 | 0.04 |
|  | Patch area +Built_up+SI + Woodlands | 6 | -175.50 | 366.66 | | 5.22 | 0.03 |
|  | Built_up+SI + Woodlands | 5 | -177.21 | 366.92 | | 5.48 | 0.02 |
|  | Patch area + Built_up | 4 | -178.76 | 367.12 | | 5.67 | 0.02 |
|  | *Male* | | | | | | |
|  | Patch area + SI | 4 | -174.71 | 359.00 | | 0 | 0.43 |
|  | Patch area +SI + Built_up | 5 | -174.40 | 361.30 | | 2.29 | 0.14 |
|  | Patch area + SI + Woodlands | 5 | -174.67 | 361.84 | | 2.83 | 0.10 |
|  | Built_up+ SI | 4 | -176.28 | 362.17 | | 3.16 | 0.09 |
|  | Built_up | 3 | -178.37 | 363.67 | | 4.66 | 0.04 |
|  | SI | 3 | -178.42 | 363.76 | | 4.75 | 0.04 |
|  | Patch area + Built_up + SI + Woodlands | 6 | -174.22 | 364.10 | | 5.09 | 0.03 |
|  | Built_up + SI + Woodlands | 5 | -176.15 | 364.80 | | 5.80 | 0.02 |
|  | Null | 2 | -180.35 | 365.14 | | 6.13 | 0.02 |
|  | Patch area | 3 | -179.13 | 365.17 | | 6.16 | 0.02 |
| *Sedentary species* | *Female* | | | | | | |
|  | Patch area + SI | 4 | -176.77 | 363.14 | | 0 | 0.34 |
|  | Patch area +SI + Woodlands | 5 | -176.44 | 365.39 | | 2.25 | 0.11 |
|  | Patch area +SI + Built_up | 5 | -176.62 | 365.74 | | 2.60 | 0.09 |
|  | Built_up | 3 | -179.67 | 366.26 | | 3.12 | 0.07 |
|  | Built_up +SI | 4 | -178.46 | 366.52 | | 3.38 | 0.06 |
|  | Patch area | 3 | -179.86 | 366.64 | | 3.51 | 0.06 |
|  | Null | 2 | -181.26 | 366.95 | | 3.82 | 0.05 |
|  | SI | 3 | -180.09 | 367.11 | | 3.97 | 0.05 |
|  | Patch area +Built_up+SI + Woodlands | 6 | -176.01 | 367.68 | | 4.54 | 0.03 |
|  | Built_up + Woodlands | 4 | -179.11 | 367.81 | | 4.67 | 0.03 |
|  | Patch area + Built_up | 4 | -179.24 | 368.07 | | 4.94 | 0.03 |
|  | Built_up + SI + Woodlands | 5 | -177.97 | 368.44 | | 5.31 | 0.02 |
|  | *Male* | | | | | | |
|  | Patch area + SI | 4 | -176.21 | 362.01 | | 0 | 0.38 |
|  | Patch area +SI + Woodlands | 5 | -175.95 | 364.39 | | 2.39 | 0.12 |
|  | Patch area + SI + Built_up | 5 | -176.00 | 364.50 | | 2.49 | 0.11 |
|  | Built_up+ SI | 4 | -177.97 | 365.53 | | 3.52 | 0.07 |
|  | Built_up | 3 | -179.44 | 365.80 | | 3.79 | 0.06 |
|  | Patch area | 3 | -179.81 | 366.53 | | 4.53 | 0.04 |
|  | Patch area + Built_up + SI + Woodlands | 6 | -175.45 | 366.56 | | 4.55 | 0.04 |
|  | SI | 3 | -179.85 | 366.62 | | 4.62 | 0.04 |
|  | Null | 2 | -181.25 | 366.93 | | 4.92 | 0.03 |
|  | Built_up + Woodlands | 4 | -178.93 | 367.46 | | 5.45 | 0.03 |
|  | Built_up + SI + Woodlands | 5 | -177.53 | 367.56 | | 5.55 | 0.02 |

**Table S5**. Model-averaging results (from the model set with cumulative Akaike weights ≤ 0.95) for all species, and best-supported model results for passerine and sedentary species, examining the variation in residual achromatic plumage color (accounting for species richness) in relation to landscape and habitat metrics for female and male birds. Metrics included patch area, shape index (SI), the cover percentage of woodlands within patches (Woodlands), and the percentage of built-up areas within a 500 m buffer surrounding habitat patches (Built_up). Patch area and SI were log-transformed, and all variables were centered and standardized. Statistics include regression coefficient estimates, standard errors (*SE*), *t* values, and *p* values.

| Group | Term | Estimate | *SE* | *t* | *p* |
| --- | --- | --- | --- | --- | --- |
| *All species* | *Female* | | | | |
|  | Patch area | 50.20 | 28.51 | 1.761 | 0.078 |
|  | SI | -54.11 | 26.66 | 2.029 | 0.042 |
|  | Built_up | -45.10 | 28.66 | 1.574 | 0.116 |
|  | Woodlands | 11.48 | 26.20 | 0.438 | 0.661 |
|  | *Male* | | | | |
|  | Patch area | 51.25 | 28.22 | 1.816 | 0.069 |
|  | SI | -56.22 | 26.22 | 2.144 | 0.032 |
|  | Built_up | -44.87 | 28.74 | 1.561 | 0.119 |
|  | Woodlands | 10.89 | 25.68 | 0.424 | 0.671 |
| *Passerine species* | *Female* | | | | |
|  | Patch area | 45.43 | 17.94 | 2.530 | 0.018 |
|  | SI | -48.69 | 17.94 | -2.713 | 0.012 |
|  | *Male* | | | | |
|  | Patch area | 47.44 | 17.23 | 2.753 | 0.010 |
|  | SI | -52.42 | 17.23 | -3.042 | 0.005 |
| *Sedentary species* | *Female* | | | | |
|  | Patch area | 47.78 | 18.46 | 2.590 | 0.015 |
|  | SI | -45.89 | 18.46 | -2.486 | 0.019 |
|  | *Male* | | | | |
|  | Patch area | 49.39 | 18.12 | 2.730 | 0.011 |
|  | SI | -49.04 | 18.12 | -2.707 | 0.012 |

**Table S6.** Results of spatial autocorrelation models examining the variation in residual achromatic plumage color (accounting for species richness) in relation to landscape and habitat metrics for female and male birds across all, passerine and sedentary species. Only the significant predictors from the prior best-fitting or averaged models were included. Statistics include coefficient estimates, standard errors (*SE*), *v* values, and *p* values.

| Group | Term | Estimate | *SE* | *v* | *p* |
| --- | --- | --- | --- | --- | --- |
| *All species* | *Female* | | | | |
|  | Intercept | -4.72 | 19.30 | -0.24 | 0.807 |
|  | Area | 88.35 | 21.50 | 4.11 | <0.001 |
|  | SI | -89.45 | 21.27 | -4.21 | <0.001 |
|  | *Male* | | | | |
|  | Intercept  Area | -4.87  90.47 | 19.27  21.51 | -0.25  4.21 | 0.801  <0.001 |
|  | SI | -91.30 | 21.26 | -4.29 | <0.001 |
| *Passerine species* | *Female* | | | | |
|  | Intercept | -3.17 | 14.43 | -0.22 | 0.826 |
|  | Area | 56.89 | 16.17 | 3.52 | <0.001 |
|  | SI | -59.15 | 15.97 | -3.70 | <0.001 |
|  | *Male* | | | | |
|  | Intercept | -2.89 | 14.19 | -0.20 | 0.838 |
|  | Area | 57.40 | 16.00 | 3.59 | <0.001 |
|  | SI | -61.44 | 15.76 | -3.90 | <0.001 |
| *Sedentary species* | *Female* | | | | |
|  | Intercept | -2.34 | 13.83 | -0.17 | 0.865 |
|  | Area | 60.58 | 15.40 | 3.93 | <0.001 |
|  | SI | -60.47 | 15.24 | -3.97 | <0.001 |
|  | *Male* | | | | |
|  | Intercept | -2.80 | 13.74 | -0.20 | 0.839 |
|  | Area | 62.25 | 15.33 | 4.06 | <0.001 |
|  | SI | -63.40 | 15.16 | -4.18 | <0.001 |


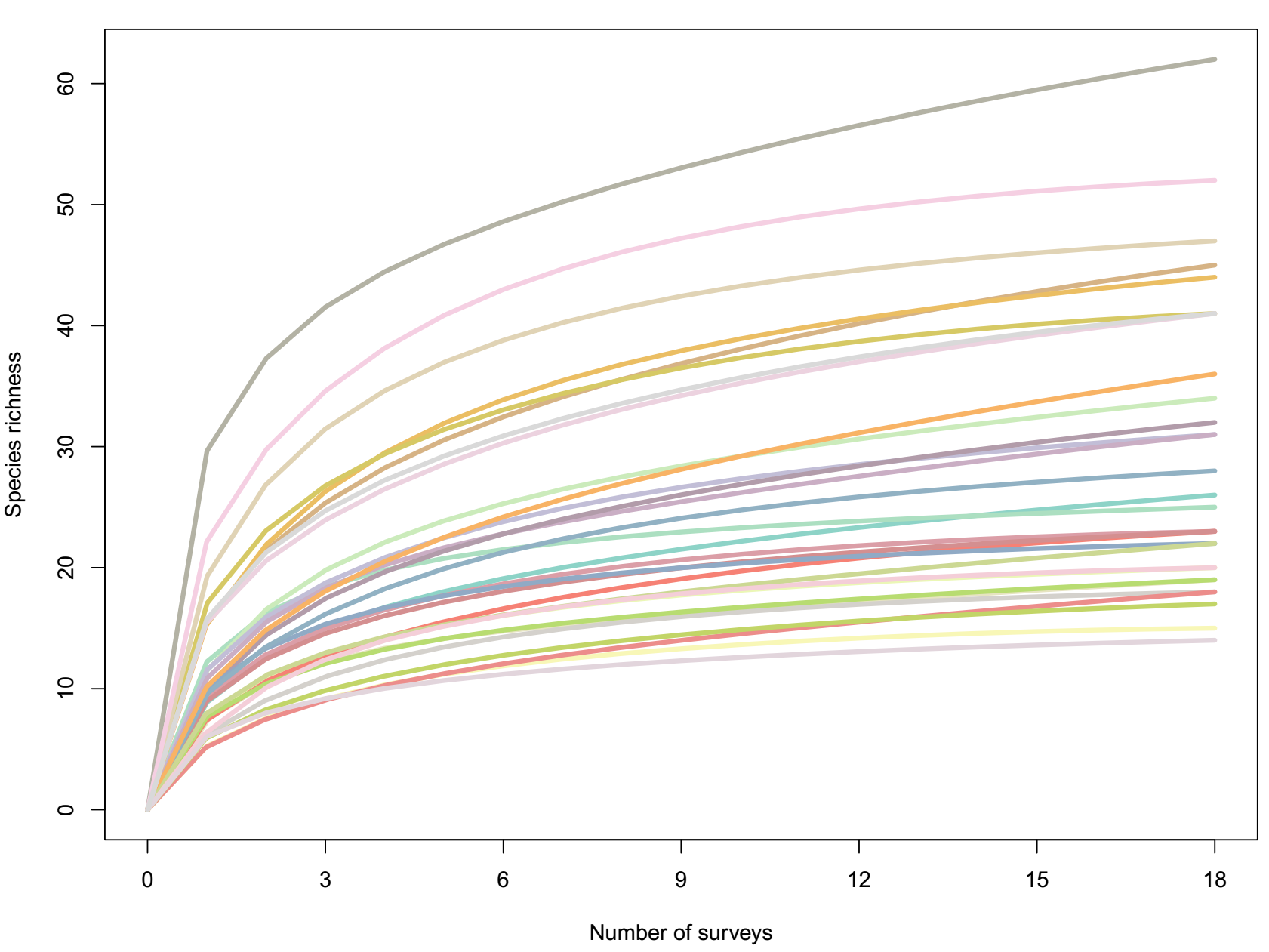


**Figure S1.** Sample-based curves of bird species in 30 patches.
